# From Affect to Values: A Lexical Approach

**DOI:** 10.1111/jopy.70022

**Published:** 2025-09-17

**Authors:** Xi Chen, Shengquan Ye

**Affiliations:** ^1^ Department of Behavioural and Social Sciences City University of Hong Kong Hong Kong

**Keywords:** affect, Chinese Personal Values Dictionary, CPVD, lexical analysis, personal values, value development

## Abstract

**Introduction:**

Personal values act as guiding principles in life and are thought to be connected to affective experiences; however, past research has primarily examined the direction from values to affect rather than the reverse. This study identified theoretical frameworks suggesting a causal pathway from affect to values and tested this pathway using a lexical approach.

**Methods:**

Study 1 (*N* = 230) developed and validated a Chinese Personal Values Dictionary (CPVD) to assess personal values in Chinese texts, revealing meaningful correlations between self‐report values and those identified through the CPVD. Using the CPVD, Study 1 also investigated the relationship between past affect and values with cross‐sectional data (*N* = 230), while Study 2 analyzed real‐time panel data from social media (*N* = 14,020) during the COVID‐19 pandemic.

**Results:**

Results indicated that individuals with positive affect tended to prioritize anxiety‐free values (openness to change and self‐transcendence), suggesting that positive affect fosters a commitment to the greater good, independence, novelty, and personal growth. In contrast, anxiety‐related values (conservation and self‐enhancement) displayed a more intricate relationship with affective experiences, indicating that the mechanisms underlying value development extend beyond mere anxiety‐related factors.

**Conclusion:**

This research offers valuable insights into how affective experiences contribute to value development through a lexical approach.

## 1 Introduction

Serving as guiding principles, personal values influence our attitudes, decisions, and behaviors (Rokeach 1973; Schwartz 1992). Why do we prioritize some values over others? One possible explanation for the individual differences in value importance is affect, which reflects our emotional state and overall well‐being.

Affect is related to value development in various ways. On one hand, the Theory of Basic Human Values (Schwartz 1992, 2016a) identifies affect, particularly the unpleasant feeling of anxiety, as a source of the value structure. Some values (i.e., conservation and self‐enhancement) are developed to cope with anxiety, while others (i.e., openness to change and self‐transcendence) emerge in its absence. On the other hand, a motivational perspective suggests that affect provides feedback to our sensory system, signaling the presence of rewards or punishments in the external environment (Frijda 1988; Lang and Bradley 2010). This feedback promotes the pursuit of values aligned with motivational goals, either to seek rewards or avoid punishments.

Despite its theoretical plausibility, research has largely neglected how everyday affect might shape the goals we aspire to pursue. Limited studies examining the influence of affect on values have used either cross‐sectional data (e.g., Sagiv and Schwartz 2000; Sortheix and Lönnqvist 2015) or panel data (e.g., Fischer and Karl 2023; Williams et al. 2015), but the mixed results hinder clear conclusions.

As an alternative to traditional self‐report measures, the present study aimed to develop a text‐based measure of personal values in Chinese and examined past affect's role in value development using a lexical approach, comparing results with self‐report measures. The lexical approach offers an objective, efficient alternative for self‐report measures, allowing real‐time analysis with larger, diverse samples (Pennebaker et al. 2003). Using a lexical approach in this research could provide novel insights into the role of affect in value development.

### 1.1 The Concept and Structure of Values

We begin by introducing the widely accepted concept and structure of personal values, as well as the sources of value distinctions, using the Theory of Basic Human Values (Sagiv and Schwartz 2022; Schwartz 1992). This theory defines values as desirable goals that guide our attitudes and behaviors. Values are ranked by importance, meaning that the same individual may prioritize values differently based on their relative significance. This relative importance forms a framework of desirable goals, guiding an individual's cognition and behaviors across situations and over time (Skimina et al. 2018).

**FIGURE 1:**
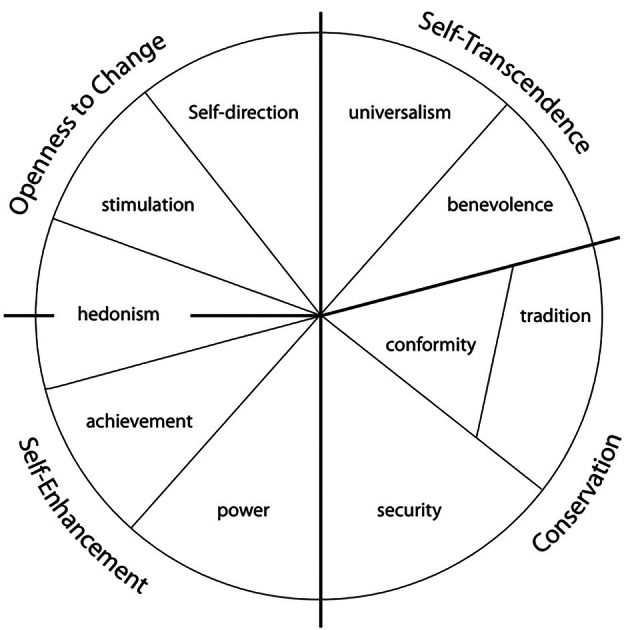
The circular continuum of values. The figure was adapted from Schwartz (2012).

Due to the degree of compatibility and conflict among the goals that values express, they form a circular continuum (see Figure 1), in which adjacent values (e.g., stimulation and self‐direction) share compatible goals, whereas more distant values (e.g., power and universalism) reflect more conflicting goals. The circular continuum of human values leads to 10 basic value types and four higher‐order value dimensions (Schwartz 1992). The four higher‐order value dimensions include openness to change (OTC), self‐transcendence (ST), conservation (CON), and self‐enhancement (SE). OTC covers goals related to independent intellectual and emotional interests toward uncertainty, encompassing the basic values of hedonism, stimulation, and self‐direction. ST refers to goals that transcend selfish concerns and promote the welfare of others, covering the basic values of universalism and benevolence. CON refers to goals for preserving the status quo and ensuring certainty in close relationships, institutions, and traditions, covering the basic values of conformity, tradition, and security. SE refers to goals that promote personal interests, potentially at the expense of others, covering the basic values of power and achievement.

To present a comprehensive yet concise relationship between values and affect, this research primarily discusses the four higher‐order values, while the results of the 10 basic values are discussed in detail when necessary.

### 1.2 Affect as a Source of Relative Value Hierarchies

People prioritize values based on their affective experiences. As outlined by Schwartz (2016a), anxiety is a key source of distinctions between anxiety‐based and anxiety‐free values. Anxiety‐based values (SE^1^ and CON) arise from motivations to control or avoid anxiety, are argued to be prioritized by individuals dissatisfied with their lives due to insecurity or uncertainty (Schwartz and Sortheix 2018). In contrast, anxiety‐free values (OTC and ST) reflect growth and self‐expansion, and are argued to be prioritized by individuals who are satisfied with life, as these people possess the emotional resources necessary to pursue these goals.

Affective experiences may also influence values based on the motivations to approach reward or avoid punishment (Fischer and Karl 2023). Affect reflects how we are doing (Tamir et al. 2016), signaling potential rewards and threats in the environment. People rely on their current feelings heuristically, allowing these feelings to inform their judgments and decisions (Schwarz and Clore 1983; Zeelenberg et al. 2008). Depending on the emotional responses, individuals may adjust their goals either by maintaining contact with or distancing themselves from a subject or event (Frijda et al. 1989; Scherer and Moors 2019). The motivational perspective of affect highlights its role in prompting responses to seek rewards or avoid punishment (Lang and Bradley 2010). Research has established a relationship between motivational direction and the pleasantness (vs. unpleasantness) of affect, linking positive affect to reward‐seeking and negative affect to punishment avoidance (Campbell et al. 2023; Gable and Harmon‐Jones 2013).

Relatedly, Schwartz (2016a) has connected anxiety‐based and anxiety‐free values to recognized motivational theories about seeking rewards and avoiding punishment. Higgins (1997) identified two basic regulatory systems: one focused on preventing loss and punishment, and another focused on achieving rewards and gains. The anxiety distinction in values reflects similar motivational foundations. Anxiety‐based values, which developed to manage anxiety, are linked to the system regulating punishment avoidance, whereas anxiety‐free values, which orient toward self‐expansion and growth, are more associated with the system that regulates reward pursuit (Leikas et al. 2009; Schwartz 2006).

The alignment of values and affect in terms of motivations to seek reward and avoid punishment suggests a coherent framework for understanding the influence of affect on values. Given the compatible motivations to approach rewards, we hypothesize that past experiences of positive affect will promote anxiety‐free values. Due to the compatible motivations to prevent loss, we hypothesize that past experiences of negative affect will promote anxiety‐based values.

### 1.3 Empirical Findings About the Influence of Affect on Values

Despite its theoretical plausibility, most studies on the relationship between personal values and affect have focused on the reverse causal pathway, exploring how values influence our desired feelings (e.g., Tamir et al. 2016; Tsai et al. 2007) and actual feelings (e.g., Brosch et al. 2011; Conte et al. 2023; De Leersnyder et al. 2018). Evidence on how past affect shapes personal values remains limited. Existing research on value development has primarily tested the interplay between well‐being and values (e.g., Fischer and Karl 2023; Oppenheim‐Weller et al. 2018; Williams et al. 2015). As a critical component of subjective well‐being, affective well‐being—specifically focusing on happiness and sadness (Boer 2017)—could provide valuable insights for our research about affective experiences.

Studies examining the interplay between well‐being and values have used both cross‐sectional data (e.g., Hanel et al. 2023; Sagiv and Schwartz 2000; Sortheix and Lönnqvist 2015) and panel data (e.g., Fischer and Karl 2023; Williams et al. 2015), but mixed results limit the ability to draw clear conclusions. Among those using cross‐sectional data, some findings align with our hypotheses. For example, Part I of Sagiv and Schwartz (2000) tested the relationship between recalled affective well‐being (over the past few weeks) and values. Their results showed positive correlations between positive affect and stimulation, self‐direction, and achievement, and negative correlations between positive affect and security, conformity, and tradition. However, results from other studies are mixed. Sortheix and Lönnqvist (2015) collected cross‐sectional data on recalled affect from student samples in Argentina, Bulgaria, and Finland, finding generally no relationship between past affective experiences and values across the samples. Similarly, Hanel et al. (2023) tested the interplay between recalled affect over the past month and values using cross‐sectional data from Europe/Schengen, Turkey, and India. Their results indicated small correlations (ranging from −0.09 to 0.20) between affective well‐being (including anxiety, stress, negative affect, and positive affect) and basic values.

Another line of research has focused on the mutually reinforcing (or bi‐directional) relationship between values and subjective well‐being over time, using panel data collected repeatedly from the same individuals. However, recall biases and the limited discussion of specific value and affect types restrict clear conclusions. For instance, Fischer and Karl (2023) examined the interplay between overall well‐being and values using self‐report panel data from daily diaries. Their network analysis revealed a positive time‐lagged effect of ST on well‐being, but no reverse effect of well‐being on any values. A limitation is that they calculated an overall well‐being score using the Warwick–Edinburgh Mental Well‐being Scale, which encompasses both affective and life satisfaction aspects, without further discussing the effect of affective well‐being on values. In addition, the affective experiences recalled at the end of each day are subject to recall biases that can distort memory retrieval (Dejonckheere and Erbaş 2021, 64–65).

In a similar vein, Williams et al. (2015) measured adolescents' self‐reported well‐being (affect and life satisfaction) and values over 1 year. Their results showed that higher baseline life satisfaction predicted greater value importance on average, but baseline positive and negative affect did not significantly relate to average value importance over the year. A limitation of their study is the focus on average value importance without analyzing affect's relationship with each value type. Also, recalled well‐being may again be subject to recall biases.

Overall, existing studies on well‐being and values yield mixed and inconclusive results, limited by recall biases of affect and insufficient discussion on specific value and affect types. In contrast to existing research, the present research examines the influence of positive and negative affect on four higher‐order values using cross‐sectional (Study 1, Phase 3) and panel data (Study 2). To reduce recall bias, Study 2 used lexical analysis of real‐time texts from participants in natural contexts. By integrating mixed data sources and study designs, this research offers nuanced insights into affect's role in value development.

### 1.4 A Lexical Approach to Psychological Concepts

People convey and understand each other's internal thoughts and feelings through language (Iliev et al. 2015; Tausczik and Pennebaker 2010). A lexical approach examines psychological concepts in language using lists of words extracted from dictionaries (Angleitner et al. 1990). Over the past century, psychologists have used this approach to study psychological constructs, including personality (e.g., McCrae and Costa 1985; Oreg et al. 2020), affect and emotions (e.g., Abplanalp et al. 2017; Barrett 2004), morality (e.g., Graham et al. 2009), and personal values (e.g., Boyd et al. 2021; De Raad et al. 2016; Ponizovskiy et al. 2020).

The lexical approach is based on the assumption that more significant characteristics are more likely to be expressed in language (Goldberg 1981). Consequently, word counting often serves as the starting point for many psychological studies using lexical analysis, providing a basic quantitative measure of language use. A lexical analysis begins with a list of words from lexicons (i.e., dictionaries) relevant to a particular concept (De Raad et al. 2016). By counting the frequency and distribution of words used by an individual or within a given text, researchers can identify patterns and extract initial insights about the individual's psychological characteristics, cognitive processes, or emotional states. While word counting is a crucial first step in understanding psychological concepts, lexical analysis in psychology extends beyond word counting, integrating techniques such as word embedding, deep learning, and large language models (Bao 2024), each offering a distinct perspective on language data.

Of particular interest to this research, we identify several advantages of using lexical analysis to test the role of affect in value development. First, lexical analysis based on word counting can provide more objective information than self‐report measures. Traditionally, measures of affect and personal values have relied heavily on self‐reports, which are susceptible to social desirability (where participants provide responses they believe are socially acceptable) and self‐delusion (where individuals hold inaccurate or distorted beliefs about themselves; Lechner et al. 2024; Quirin and Bode 2014). In contrast, lexical analysis using a word‐counting approach is more objective because it relies on observable and quantifiable linguistic patterns rather than subjective self‐assessment (Pennebaker et al. 2003; Schwartz et al. 2013).

Second, lexical analysis is time‐ and resource‐efficient for studying the influence of affect on values using panel data (Pennebaker et al. 2003; Tausczik and Pennebaker 2010). Existing studies mainly rely on time‐intensive, costly self‐report methods like daily diaries and longitudinal designs (Wright and Markon 2016, 424–425), which face recall biases, meaning that recalled memories of past affective experiences may be distorted (e.g., due to knowing the actual outcomes at later time points; Dejonckheere and Erbaş 2021, 65).

Lexical analysis also enhances accessibility across diverse populations and text sources (e.g., online contents, transcripts of spoken text, and text corpora), allowing for greater replication potential in cross‐cultural comparisons compared to traditional methods limited by sample constraints (Pennebaker et al. 2003).

In the present research, we used a word‐counting approach to understand the role of past affect on values in Chinese.^2^ As a key premise for word counting, lexicons in the target language are essential. Several lexicons of affect are available in Chinese, including the Chinese Sentiment Lexicon for Internet (CSLI; Zhao et al. 2019), the Chinese Valence‐Arousal Words (CVAW; Yu et al. 2016), and the Chinese Linguistic Inquiry and Word Count (Chinese LIWC; Huang et al. 2012). We chose CSLI for analyzing affect as it was developed recently using modern Chinese corpora (e.g., Sina Weibo and SogouW Internet Lexicon) and has shown good convergence with human‐rated affect scores (*r* = 0.70 for valence and *r* = 0.59 for arousal). Compared with CVAW and Chinese LIWC, which were validated with Taiwan Chinese raters, CSLI was developed based on indigenous mainland Chinese language and is well‐suited for analyzing social media texts created by mainland Chinese users.

However, available value lexicons exist mainly for English and other European languages (e.g., De Raad et al. 2016; Ponizovskiy et al. 2020; Renner 2003). A theory‐driven lexicon for personal values in Chinese is scarce. To use a lexical approach to measure personal values in the Chinese language, we translated a theory‐driven personal values dictionary (PVD; Ponizovskiy et al. 2020) developed based on Schwartz's theory of basic values from English to Chinese and provided preliminary validation results (Study 1, Phase 1, and Phase 2). We refer to the translated lexicon of personal values as the Chinese Personal Values Dictionary (CPVD).

### 1.5 Current Project

Taken together, based on different theories, we hypothesize that anxiety‐free, reward‐approaching values associate with past positive affect, while anxiety‐based, punishment‐avoiding values associate with past negative affect. The aim of the current research is twofold. First, to measure affect and values in Chinese text, we developed and preliminarily validated a theory‐driven personal values dictionary in Chinese by translating the original personal values dictionary (Study 1, Phase 1 and Phase 2). Second, to examine affect's role in value development, we collected data using cross‐sectional and repeated measures designs, using either self‐report measures or lexical approaches. Specifically, we tested the relationship between recalled affect over the past month and values using cross‐sectional data from self‐report measures and lexical analysis of writing tasks (Study 1, Phase 3). We then applied a lexical approach to investigate the interplay between affect and values using panel data from real‐life social media (Study 2).

## 2 Study 1

Study 1 comprises three phases of work. To examine the influence of affect on values using a lexical approach in the Chinese language, Study 1, Phase 1 developed a theory‐driven Chinese personal values dictionary (CPVD). Study 1, Phase 2 then provided preliminary validation results. Study 1, Phase 3 aimed to test the role of recalled affect in value development using a cross‐sectional design similar to existing studies (e.g., Sagiv and Schwartz 2000; Sortheix and Lönnqvist 2015). Data from both self‐reports and a lexical approach were analyzed.

### 2.1 Phase 1: Developing the Chinese Personal Values Dictionary

Following Schwartz's cross‐culturally validated circular structure of basic values (e.g., Schwartz and Bilsky 1990; Schwartz et al. 2012; Skimina et al. 2018), we developed CPVD by translating the original PVD (Ponizovskiy et al. 2020) from English to Chinese. A translation approach mirrors development methods of established lexicons (e.g., the Traditional Chinese version of LIWC; Huang et al. 2012) and ensures comparability of performance with prior work.

**FIGURE 2:**
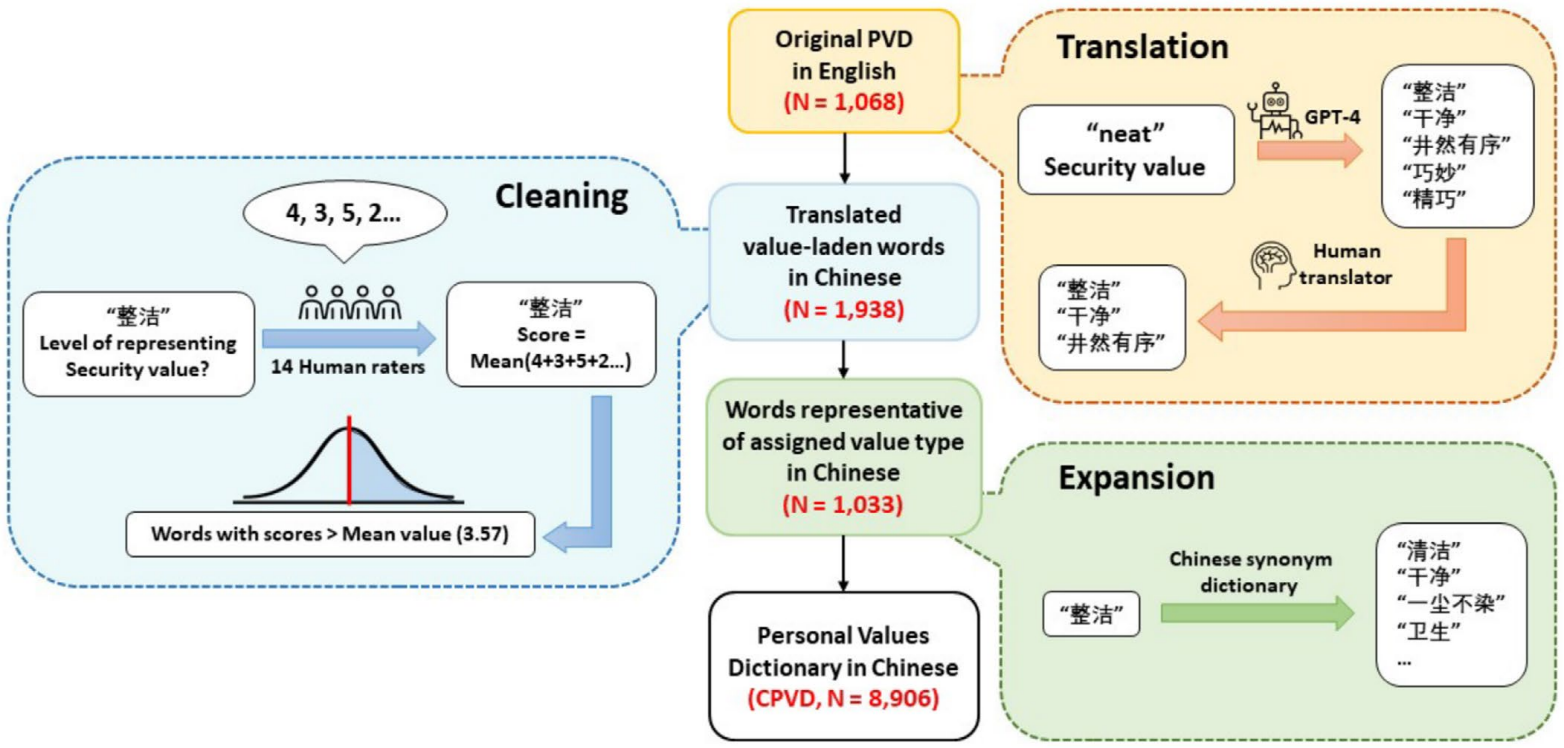
Process of developing the CPVD (Study 1, Phase 1).

The procedure for developing CPVD involved three steps: Translation, cleaning, and expansion, as illustrated in Figure 2.

#### 2.1.1 Translation

PVD contains 1068 value‐laden words in English, covering the 10 basic value types. The translation was performed by two translators: an AI chatbot based on the GPT‐4 model (OpenAI 2023) and a human translator familiar with personal values. We chose GPT‐4 for its efficiency in handling bulk translations, such as the 1068 words in the PVD, and its high accuracy in translating texts into Mandarin Chinese (80.1%; OpenAI 2023).

Initially, we instructed GPT‐4 to translate each value‐laden word in PVD to up to five Chinese words based on its original value type. The human translator then reviewed the list generated by GPT‐4, removing inappropriate translations and adding more appropriate ones.^3^ The human translator also reviewed the translation according to four established rules (Huang et al. 2012; Renner 2003). First, repeated words within the same value category, which mainly occurred due to different tenses or synonyms (e.g., “avoid” and “avoided” both translate to “避免” in Chinese), were removed. Second, words with undesirable meanings were excluded (e.g., “大灾难” translated from “catastrophic”; “暴力” translated from “violence”), as they do not align with the definition of values related to desirable goals. Third, words representing psychological concepts other than values—such as emotions (e.g., “不安” translated from “qualms”), personal characteristics (e.g., “谨慎” translated from “careful”), and societal roles (e.g., “民主党人” translated from “democrats”)—were removed.

The whole translation process eventually resulted in a list of 1938 Chinese words covering the 10 basic value types for further refinement.

#### 2.1.2 Cleaning

To ensure that the translated words closely aligned with their corresponding value types, 14 native Chinese speakers volunteered to rate how well each translated word matched its designated value type. Prior to the rating task, the raters were introduced to the concept of personal values and the definitions of the 10 basic value types. A customized data collection program was developed using MATLAB App Designer (MathWorks 2024) to facilitate the rating process. For each word, raters were presented with the Chinese translation and its assigned value type, evaluating how well the word represented its value type on a Likert scale from 1 (very low) to 5 (very high). To ensure data quality, 20 attention check questions were included; data from 10 raters who correctly answered over 80% of attention check questions were left for analysis.

We evaluated the performance of the translated words by examining the mean score for each word across all raters. The overall mean score across raters and words was 3.57 (SD = 0.68). We kept 1033 words scoring above the mean, ensuring that the selected words were strongly representative of their assigned value type.

#### 2.1.3 Expansion

To enrich the lexicon with more indigenous language content, we expanded the 1033 words by extracting synonyms from a Chinese synonym dictionary called “HIT IR‐Lab Tongyici Cilin (Extended)” (Che 2010), hereinafter referred to as “Cilin.”^4^ This dictionary contains 77,343 words organized by meaning. For each value‐laden word, we extracted all synonyms from Cilin. For example, synonyms like “身强体壮” and “健硕” were added for the value‐laden word “健康” (“health”). This expansion process resulted in a total of 8906 words in Chinese, covering the 10 basic value types. The expanded value dictionary of 8906 words was named the Chinese Personal Values Dictionary (CPVD).^5^

### 2.2 Phase 2: Validating the Chinese Personal Values Dictionary

#### 2.2.1 Participants and Procedure

After developing the CPVD, we tested its performance by comparing value scores calculated from texts using lexical analysis (text‐based values) with the self‐report values of the same individuals. We recruited native Chinese speakers through Credamo, an online survey platform. To estimate the required sample size, we conducted a power analysis using G*Power (Faul et al. 2009). Previous studies reported a correlation between self‐report values and text‐based values ranging from −0.07 to 0.33 (Fischer et al. 2022; Ponizovskiy et al. 2020). We used an effect size of 0.20 for the power analysis, which indicated a sample size of 193 for a power of 0.80.

In the survey, participants first completed questionnaires and writing tasks regarding their values and affective experiences in the past month. The writing task asked participants to write short paragraphs about their experiences, including information about values, behaviors, or feelings, which is commonly used to measure text‐based values (Boyd et al. 2021; Fischer et al. 2022). Measures of affect were included to test the interplay between past affect and values. Two attention check questions were integrated into the questionnaire. Upon completing the survey, participants received 10 RMB as compensation for their time and effort.

To estimate the time required for the writing tasks, 30 participants were invited to complete the questionnaire in a pilot test on Credamo. In the main round of data collection, 200 participants were recruited. All participants answered the attention check questions correctly, resulting in data from *N* = 230 participants (64.78% female, *M*_age_ = 32.00) for validation analysis.

#### 2.2.2 Materials

##### 2.2.2.1 Text‐Based Values

To measure text‐based values, participants completed two writing tasks adapted from previous research (Boyd et al. 2021; Fischer et al. 2022). In the first task, participants wrote about their core guiding principles in life, reflecting on how they make important decisions, interact with others, and determine what matters to them and those around them. In the second task, participants described activities they desired to pursue, including daily activities, hobbies, and aspirational pursuits. For each task, participants had 1 min to think about the content before writing a passage of at least 150 characters within 5 min. They were encouraged to write continuously after starting. The texts from both tasks were combined for analyzing an individual's text‐based values.

**FIGURE 3:**
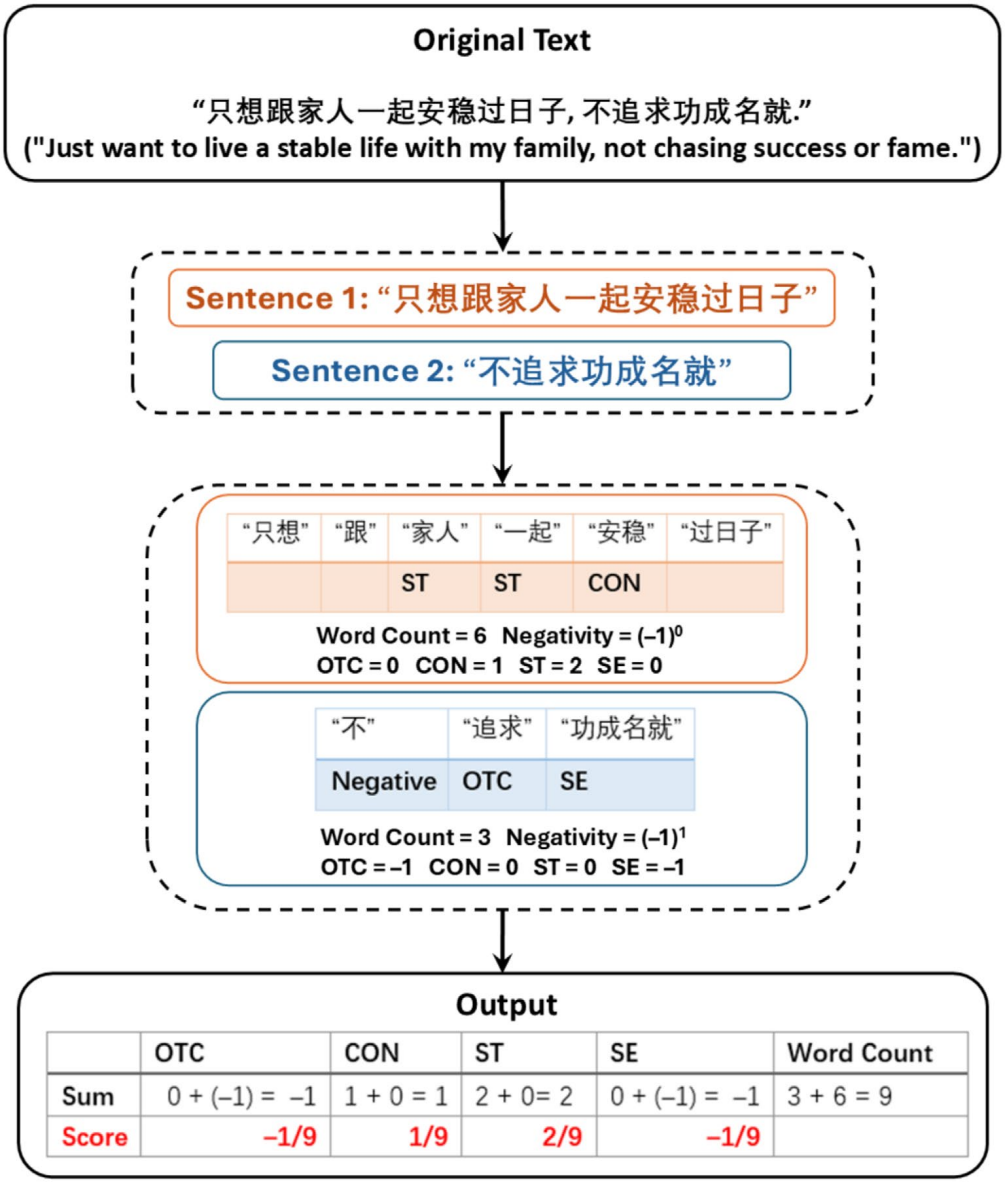
Procedure of calculating text‐based value scores (Study 1, Phase 2).

To calculate text‐based value scores, we used the CPVD for word counting. As words in Chinese are not separated by spaces as in English, we segmented the passages into words using the *jiebaR* package in R (Qin and Wu 2022). Figure 3 illustrates the procedure of calculating text‐based value scores.

Unlike previous studies, we weighted word frequencies by the positive and negative tone of the context. Previous methods measured text‐based values using raw word frequencies (e.g., Fischer et al. 2022) and the proportion of words in a value category relative to the total quantity of value‐laden words (Ponizovskiy et al. 2020), which could lead to biased results by neglecting contextual negativity. For example, the phrases “我重视为人正直” (“I think integrity is important”) and “我不重视为人正直” (“I don't think integrity is important”) would both yield a score of 1 for conformity due to the presence of the conformity‐laden word “正直” (“integrity”). However, the negative context of the latter sentence contradicts the conformity value.

To mitigate this bias, we segmented large paragraphs into individual sentences based on punctuation marks. We then counted the number of negation words in each sentence.^6^ Sentences with an odd number of negation words were classified as having a negative tone, whereas those with an even number were classified as having a positive tone. For sentences with a negative tone, the frequency of value‐laden words was negatively weighted. We summed the value frequency across all sentences. Following Ponizovskiy et al. (2020), we calculated the proportion of summed value frequency for each value type relative to the total words used by each participant, yielding text‐based value scores ranging from −1 to 1.

##### 2.2.2.2 Self‐Report Values

To measure self‐report values, we used 21‐Item Portrait Values Questionnaire (21‐PVQ; Schwartz and Bardi 2001). Participants indicated how similar they felt to the portrayed individual on a scale from 1 (not like me at all) to 6 (very much like me). A sample item for Universalism states: “She thinks it is important that every person in the world be treated equally. She wants justice for everybody, even for people she doesn't know.” The Chinese version of 21‐PVQ was adapted from Ye et al. (2018). We also calculated ipsatized value scores by subtracting the mean ratings from the raw value scores for each individual (Schwartz 2006).

##### 2.2.2.3 Demographic Information

Participants reported their gender, age, and education level.

#### 2.2.3 Results and Discussion

##### 2.2.3.1 Convergence Between Text‐Based and Self‐Report Values

We first examined the similarity between self‐report and text‐based values by calculating Pearson's correlations. We expected self‐report values to be positively correlated with their corresponding text‐based values. The results for both raw and ipsatized self‐report values are presented in Table 1. Table 1 also presented the correlation between text‐based values and an individual's mean rating of all values (MRAT), which serves as a proxy for response style or social desirability (Borg and Bardi 2016).

**TABLE 1:** Pearson's correlations between self‐report values and text‐based values (*N* = 230, Study 1, Phase 2).

	Text‐based values and self‐report values (raw)	Text‐based values and self‐report values (ipsatized)	Text‐based values and self‐report values (MRAT)^a^
Four higher‐order values			
Openness to change	0.17*	0.20*	0.02
Conservation	−0.01	−0.08	0.08
Self‐transcendence	0.19**	0.13	0.11
Self‐enhancement	0.15*	0.12	0.10
Ten basic values			
Security	−0.11	−0.18**	0.11
Conformity	0.08	0.08	0.04
Tradition	−0.09	−0.11	0.003
Benevolence	0.16*	0.09	0.14*
Universalism	0.16*	0.15*	0.03
Self‐direction	0.17*	0.18**	0.04
Stimulation	0.19**	0.21**	0.02
Hedonism	−0.02	0.002	−0.03
Achievement	0.14*	0.14*	0.06
Power	0.04	−0.02	0.13*

*Note:* *p < 0.05; **p < 0.01; ***p < 0.001.

^a^ MRAT (the mean rating of all values) is calculated from each individual's mean score across all 21 value items used for the self‐report value measurement.

Text‐based scores positively correlated with self‐report raw scores for OTC (*r* = 0.17), ST (*r* = 0.19), and SE (*r* = 0.15). However, the correlation for CON was non‐significant (*r* = −0.01). The text‐based scores and MRAT scores showed non‐significant correlation for the four higher‐order values, indicating minimal response style biases for text‐based scores.

To explore why some text‐based values exhibited non‐significant or negative correlations with self‐report values, we analyzed the similarity between text‐based values and self‐report raw values for the 10 basic values (Table 1, lower section). The results revealed convergence between text‐based and self‐report values for ST values, including Benevolence (*r* = 0.16) and universalism (*r* = 0.16), most OTC values, such as self‐direction (*r* = 0.17) and stimulation (*r* = 0.19), and one SE value (achievement: *r* = 0.14). In contrast, the convergence between text‐based and self‐report values was weak for CON values, including security (*r* = −0.11), conformity (*r* = 0.08), and tradition (*r* = −0.09). Of SE and OTC values, non‐significant correlations mainly stemmed from power (*r* = 0.04) and hedonism (*r* = −0.02).

The overall convergence between text‐based and self‐report values is comparable to previous studies using the original English version of the PVD, where reported correlations ranged from −0.05 to 0.33 (Ponizovskiy et al. 2020) and −0.07 to 0.25 (Fischer et al. 2022). However, it is noteworthy that the convergence between self‐report and text‐based conformity and tradition values appears lower in the current study compared to previous research using the original PVD (e.g., 0.16 for tradition and 0.15 for conformity in Fischer et al. 2022; 0.31 for tradition and 0.07 for conformity in Ponizovskiy et al. 2020). The meanings of conservation in Chinese culture may differ from those in Western cultures (e.g., Confucian/Buddhist vs. Christian beliefs; Leung 2010), which may be reflected in the lexicons. This cultural variation could explain the weak convergence between text‐based and self‐report measures for conformity and tradition.

##### 2.2.3.2 Confirmatory Multidimensional Scaling (MDS)

**FIGURE 4:**
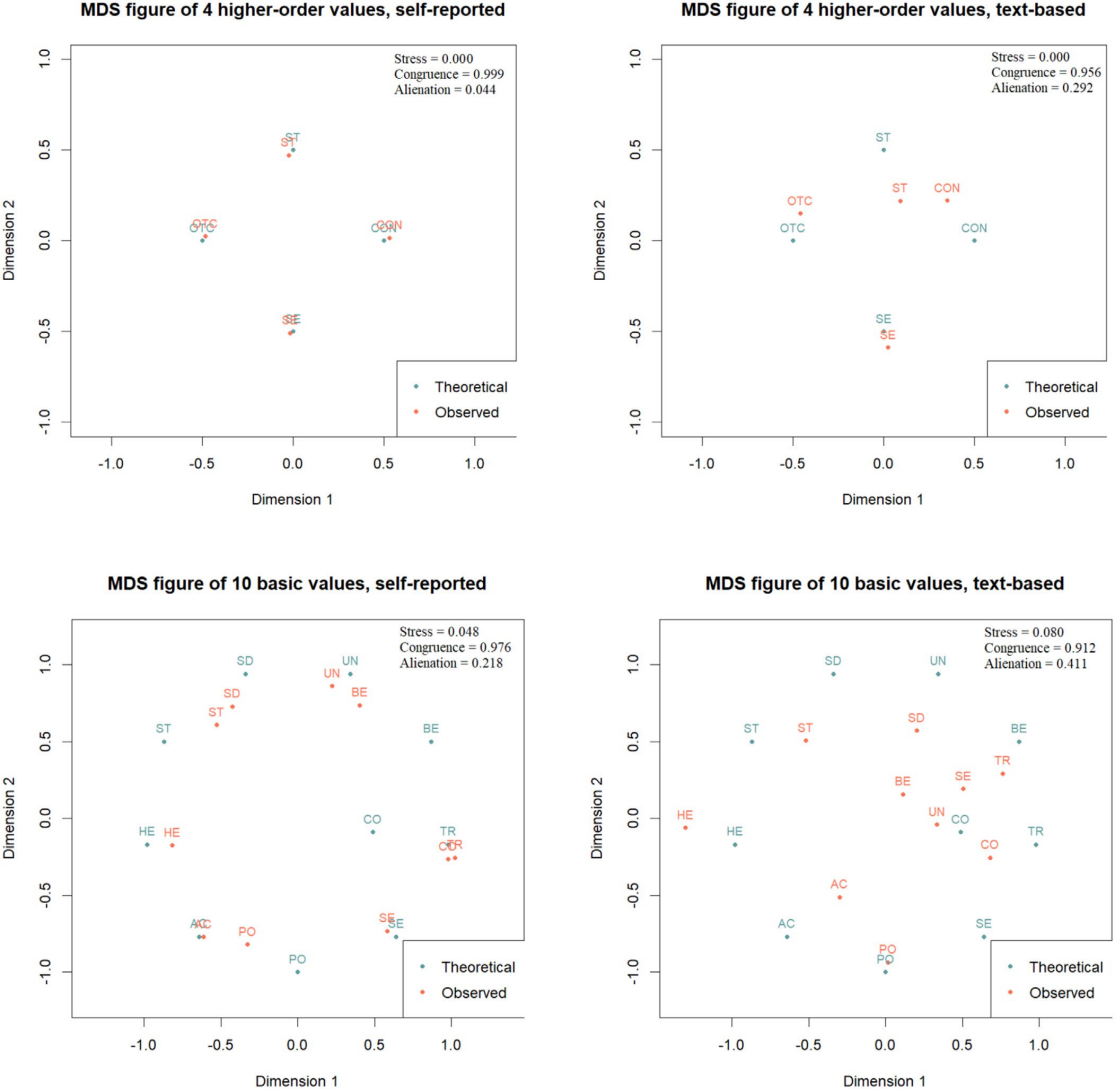
Plots of MDS analysis for the four higher‐order values and 10 basic values using text‐based scores and self‐report scores (*N* = 230, Study 1, Phase 2). In all figures, blue dots represent the theoretically expected circular structure of values, while red dots represent the observed structure of values. In the two upper figures, CON, conservation; OTC, openness to change; SE, self‐enhancement; ST, self‐transcendence. In the two lower figures, AC, achievement; BE, benevolence; CO, conformity; HE, hedonism; PO, power; SD, self‐direction; SE, security; ST, stimulation; TR, tradition; UN, universalism.

To test the theoretical circular structure for both text‐based and self‐report values, we conducted a weak confirmatory multidimensional scaling analysis using the *smacof* package in R (Mair et al. 2024). A weak confirmatory MDS imposes an ordinal configuration on the variables by predicting the variables' relative positions on a two‐dimensional space (Cieciuch 2017), enabling a comparison of the observed configuration with the theoretically expected circular structure of values (Fischer and Fontaine 2011). The MDS plots for the 4 higher‐order values and the 10 basic values, based on both text‐based scores and ipsatized self‐report scores, are presented in Figure 4. MDS goodness‐of‐fit was evaluated by the normalized stress value, with benchmarks for weak confirmatory MDS set as 0.20 (poor), 0.10 (fair), 0.05 (good), 0.025 (excellent), and 0.00 (perfect; Mair et al. 2024). To assess the similarity between the observed and theoretical configurations, we performed a Procrustes analysis and reported Tucker's congruence coefficients (Mair et al. 2024). Tucker's congruence coefficients ranging from 0.85 to 0.94 are considered “fairly similar,” while coefficients above 0.95 suggest the configurations are “equal” (Fischer and Fontaine 2011; Fischer and Karl 2019; Mair et al. 2024).

The stress values of the four MDS models ranged from 0.00 to 0.08, indicating perfect to fair model fit. The results of Procrustes analysis indicated that text‐based values exhibited similar relative positions to the theoretical configuration. Compared with the ideal theoretical structure, the four higher‐order values using text‐based scores yielded a Tucker's congruence coefficient of 0.96 (alienation coefficient = 0.29), suggesting structural similarity. Similarly, the 10 basic values using text‐based scores yielded a Tucker's congruence coefficient of 0.91 (alienation coefficient = 0.41), indicating structural similarity with the theoretical configuration.

Compared with self‐reports (Tucker's congruence = 0.999 for the 4 higher‐order values and congruence = 0.976 for the 10 basic values), the text‐based scores yielded smaller Tucker's congruence coefficients, indicating weaker alignment with the theoretical configuration. This result may stem from the conceptual connection between ST and CON in Chinese culture. For example, phrases like “孝敬父母” (filial piety to parents) blend ST (caring for others) and CON (upholding family traditions). In everyday spontaneous language, such expressions obscure distinctions between ST and CON, unlike self‐reports where explicit instructions produce clearer differentiation.

### 2.3 Phase 3: The Role of Recalled Affect on Values Using Cross‐Sectional Data in Self‐Report and Lexical Approach

#### 2.3.1 Participants

To test the influence of recalled affect on values, Study 1, Phase 3 used self‐report values and text‐based values from the same 230 participants as in Study 1, Phase 2. In addition to the value scores, we included self‐report affect and text‐based affect scores derived from another writing task completed by these participants.

#### 2.3.2 Materials

##### 2.3.2.1 Text‐Based Affect

To measure text‐based affect, participants were instructed to write a short passage describing their overall affective experiences and significant affective events from the past month. They were asked to reflect on both positive and challenging experiences, considering what happened, where it occurred, and who was involved. Participants had 1 min to think before writing a passage of at least 150 characters in 5 min and were encouraged to write continuously.

The Chinese Sentiment Lexicon for Internet (CSLI; Zhao et al. 2019) was used for affects' lexical analysis. CSLI consists of 7088 Chinese words, each annotated with a valence score (ranging from −4 to 4) and an arousal score (ranging from 0 to 8). Words with negative valence scores were classified as negative affect words, and words with positive valence were considered positive affect words. A score for text‐based positive (negative) affect was calculated as the proportion of positive (negative) affect words out of the total words used in each participant's writing passage. Unlike text‐based values, we did not weight the frequency by contextual tones, as CSLI already contains words with inherently negative meanings (e.g., “不高兴” [unhappy]).

##### 2.3.2.2 Self‐Report Affect

To measure self‐report affect, we used the Affect Valuation Index (AVI; Tsai et al. 2006). AVI comprises 39 items covering positive and negative affective experiences, varying in levels of arousal. Participants were asked to report “how much they typically felt each item on average in the past month” on a scale from 1 (never) to 5 (all the time). A Chinese version of the AVI was adapted from Tsai et al. (2006). We calculated positive (negative) affect by averaging the scores of all positive (negative) items (Clobert et al. 2022).

#### 2.3.3 Results and Discussion

Descriptive statistics for self‐report and text‐based scores are presented in Table 2. Except for CON, self‐report and text‐based scores of the same variable were positively correlated (ranging from 0.15 to 0.26), suggesting overall similarity between self‐report and text‐based measures. The correlations between all affect and value variables, both self‐report and text‐based, are reported in Table 3.

**TABLE 2:** Descriptive statistics of key variables (*N* = 230, Study 1, Phase 3).

Variables	Self‐report (raw)		Text‐based^b^		Correlation (self‐report raw and text‐based)
	Mean^a^	SD	Mean	SD	
Positive affect	3.51	0.59	29.77	6.72	0.18**
Negative affect	1.70	0.42	7.39	4.32	0.26***
Openness to change	4.46	0.71	14.28	3.60	0.17*
Conservation	4.36	0.79	13.18	3.85	−0.01
Self‐transcendence	4.97	0.64	17.85	4.65	0.19**
Self‐enhancement	4.00	0.97	9.33	2.71	0.15*

*Note:* *p < 0.05; **p < 0.01; ***p < 0.001.

^a^ The self‐report scores of affect (positive affect and negative affect) ranged from 1 to 5. The self‐report scores of four higher‐order values (openness to change, conservation, self‐transcendence, and self‐enhancement) ranged from 1 to 6.

^b^ Because the text‐based scores represent the rate of words out of total words used, they are multiplied by 100 for easier interpretations.

**TABLE 3:** Correlations between key variables (*N* = 230, Study 1, Phase 3).

Variables	1	2	3	4	5	6	7	8	9	10	11	12
Self‐report^a^												
PA	1											
2 NA	−0.55***	1										
3 OTC	0.47***	−0.28***	1									
4 CON	0.06	−0.06	−0.04	1								
5 ST	0.51***	−0.37***	0.39***	0.28***	1							
6 SE	−0.15*	0.19**	0.34***	0.26***	−0.01	1						
Text‐based												
7 PA	0.18**	−0.08	0.18**	0.11	0.19**	0.03	1					
8 NA	−0.3***	0.26***	−0.13	0.1	−0.15*	0.02	−0.36***	1				
9 OTC	0.16*	−0.03	0.17*	−0.2**	0.02	0.11	0.06	−0.13*	1			
10 CON	0.21**	−0.25***	0.07	−0.01	0.22***	−0.04	0.11	−0.1	0.3***	1		
11 ST	0.2**	−0.16*	0.09	0.03	0.19**	0.01	0.26***	−0.18**	0.39***	0.68***	1	
12 SE	0.07	−0.03	0.08	−0.01	0.06	0.15*	0.24***	−0.09	0.15*	0.23***	0.27***	1

*Note:* *p < 0.05; **p < 0.01; ***p < 0.001.

Abbreviations: CON, conservation; NA, negative affect; OTC, openness to change; PA, positive affect; SE, self‐enhancement; ST, self‐transcendence.

^a^ The self‐report scores are raw scores.

To assess whether recalled affect from the past month was related to values, we fitted multivariate models using the *lavaan* package in R (Rosseel 2024). Each model included six observed variables: two independent variables (positive affect and negative affect) and four dependent variables representing the four higher‐order values (OTC, CON, ST, and SE).^7^ Covariance was allowed between the dependent and independent variables. We tested two multivariate models with the same setup: one using self‐report scores and the other using text‐based scores. Raw scores were used for all self‐report measures to avoid linear dependence (Schwartz 2016b). We report standardized regression coefficients due to different scales of the measures across variables. We also report the same multivariate models using the 10 basic values as dependent variables (see Table S1).

We first examined the relationship between recalled affect and values using self‐report scores (see Table 4, upper section). Positive affect was positively correlated with OTC and ST, indicating that individuals who recalled more positive affective experiences in the past month reported higher levels of OTC and ST. Negative affect was only positively correlated with SE, indicating that individuals recalling more negative experiences reported higher levels of SE.

**TABLE 4:** Results of multivariate models examining the effect of recalled affect on values (*N* = 230, Study 1 Phase 3).

	*β*	SE	*p*	95% CI		*R* ^*2*^
				LL	UL	
Self‐report						
Openness to change						0.219
Positive affect	**0.448**	**0.065**	**< 0.001**	0.321	0.575	
Negative affect	−0.035	0.070	0.615	−0.172	0.102	
Conservation						0.004
Positive affect	0.037	0.079	0.642	−0.118	0.191	
Negative affect	−0.035	0.079	0.654	−0.190	0.119	
Self‐transcendence						0.275
Positive affect	**0.449**	**0.062**	**< 0.001**	0.327	0.572	
Negative affect	−0.119	0.067	0.074	−0.250	0.012	
Self‐enhancement						0.041
Positive affect	−0.066	0.077	0.392	−0.217	0.085	
Negative affect	**0.157**	**0.077**	**0.** **040**	0.007	0.308	
Text‐based						
Openness to change						0.017
Positive affect	0.014	0.070	0.836	−0.123	0.152	
Negative affect	−0.124	0.069	0.073	−0.261	0.012	
Conservation						0.016
Positive affect	0.083	0.070	0.233	−0.054	0.220	
Negative affect	−0.071	0.070	0.309	−0.208	0.066	
Self‐transcendence						0.075
Positive affect	**0.222**	**0.066**	**0.** **001**	0.091	0.352	
Negative affect	−0.101	0.068	0.136	−0.233	0.032	
Self‐enhancement						0.056
Positive affect	**0.234**	**0.067**	**< 0.001**	0.103	0.365	
Negative affect	−0.004	0.069	0.954	−0.138	0.130	

*Note:* Significant results are bolded. Raw scores are used for self‐report variables.

Next, we tested the same multivariate model using text‐based scores (see Table 4, lower section). Similar to the findings from self‐reports, positive affect correlated positively with ST, suggesting that individuals who used more positive affect words to describe their past experiences also used a greater number of ST‐related words. However, contrary to our hypothesis, positive affect showed a positive correlation with SE, indicating that individuals who used more positive affect words also used more SE‐related words.

In summary, the results of multivariate analyses partially supported our hypotheses. Specifically, self‐report data indicated that anxiety‐free values (OTC and ST) positively correlated with recalled positive affect. For anxiety‐based values, only SE was positively related to recalled negative affect. CON was unrelated to either positive or negative affect. Text‐based analysis revealed similar findings, with ST positively correlated with recalled positive affect. However, in contrast to the self‐report data, positive affect was positively associated with SE in text‐based scores. This relationship between SE and affect in text‐based scores and its divergence from self‐report data may reflect the reward‐approach motivations of Achievement (Leikas et al. 2009) or medium‐related effects in texts, which we will discuss later in the General Discussion section.

Study 1, Phase 3 used retrospectively recalled scores, which may introduce recall biases. Also, the cross‐sectional design cannot account for unobserved individual changes and provides limited causal inference (Falkenström et al. 2020). To reduce recall biases and provide insights for causal inference, Study 2 analyzed real‐life social media data to explore the link between past affect and subsequent value development.

## 3 Study 2 the Influence of Affect on Values in Real‐Life Social Media Data

To test the temporal relationship between past affective experiences and value development, Study 2 analyzed real‐time social media data collected repeatedly from the same individuals before and after the city lockdown of Wuhan, China, during the COVID‐19 pandemic. Studying how affect influences values is especially insightful during major events like life transitions (e.g., entering university) or social events (e.g., city lockdowns), as these moments intensify emotional responses that may shape values. A major event like the Wuhan lockdown likely triggered widespread affective experiences across a large population, offering a unique opportunity to examine this dynamic.

Previous research (e.g., Daniel et al. 2022) has observed significant mean‐level value change during the COVID‐19 pandemic, marked by lockdown measures (e.g., restrictions on social gatherings, requirements to work from home) that disrupted daily life. Studies also show substantial emotional responses to major pandemic events (e.g., Wuhan city's lockdown on January 23, 2020; Chen and Yik 2022), potentially driving value changes. Yet, few studies have discussed how these affective experiences influenced value development.

During the Wuhan lockdown, we predicted increases in anxiety‐based values, such as SE and CON, due to the negative emotions like anxiety and loneliness elicited by social restrictions (Palgi et al. 2020). However, increases in anxiety‐free values were also expected, as a majority of Chinese social media posts during this period reflected positive emotions like hope and encouragement (Chen and Yik 2022). Using real‐time social media data from the same individuals, Study 2 aimed to reduce recall bias and better capture how values adapt to major life events.

### 3.1 Method

#### 3.1.1 Data and Lexical Analysis

Study 2 used a secondary social media dataset named Weibo‐COV2 (Hu et al. 2020),^8^ which originally contained over 65 million posts from Sina Weibo^9^ created by 20 million active users during the COVID‐19 pandemic from December 2019 to December 2020. We extracted a panel dataset of 89,638 posts generated repeatedly by 14,092 individuals in the 2 weeks before to 2 weeks after the lockdown in Wuhan.^10^ We refer to the 2 weeks before the lockdown as Time 1 and the 2 weeks after as Time 2 for clarity.

To examine the influence of affect on values over time, we aggregated all posts in Time 1 and Time 2 to form long texts for each period, enabling us to predict individual affect and values at both time points. We removed reposted content and retained original content, eliminating data from 72 individuals with no original content, retaining 14,020 unique individuals for subsequent analysis. The same lexical analysis approach used in Study 1 was used to measure the four higher‐order value types (OTC, CON, ST, and SE) and the two affect types (positive and negative) for each individual at both time points.

#### 3.1.2 Statistical Analysis

To test the interplay between values and affect, we used cross‐lagged panel models (CLPM) to assess the relationship between affect and values while controlling for each variable's stability and time‐specific covariance. All CLPMs were estimated using the *lavaan* package in R (Rosseel 2024). Although the CLPM has faced recent criticism (see Lucas 2023), it remains useful for analyzing relationships between variables across two time points (Usami 2021).

Four CLPMs were estimated. Each CLPM included two affect variables (positive and negative affect) and one of the four higher‐order values (OTC, CON, ST, and SE) before and after the city lockdown. The models incorporated autoregressive paths to account for the stability of values and affect, and cross‐lagged paths to estimate bidirectional relationships between affect and values across Time 1 and Time 2. Our focus was on the influence of affect on values, represented by the regression coefficient of affect at Time 1 on values at Time 2. The results of the same CLPM for each basic value are reported in Tables S3–S6.

During the analysis, we noted that all variables had standard deviations larger than their means, indicating positively skewed distributions. Due to this non‐normal distribution, we used standard maximum likelihood estimation with robust standard errors for parameter estimation (Rosseel 2024). Since all CLPMs included variables at two time points and were saturated models (Usami 2021), traditional model fit indices (e.g., RMSEA) are not informative in this context. Therefore, we report standardized coefficients, standard errors, significance levels, 95% confidence intervals, and the proportion of explained variance for the Time 2 variables.

### 3.2 Results and Discussion

Table 5 reports the descriptive statistics and correlations between variables at Time 1 and Time 2. Table 6 and Table 7 summarize the CLPM results for anxiety‐free and anxiety‐based values, respectively. We first examined the stability of text‐based values across the two periods. The autoregressive coefficients were positive and significant for all variables, suggesting overall stability in the text‐based values and affect over time.

**TABLE 5:** Descriptive statistics and correlations between variables (*N* = 14,020, Study 2).

Variables	Mean^a^	SD^a^	1	2	3	4	5	6
PA	14.06	11.93	1					
2 NA	5.99	6.67	−0.01*	1				
3 OTC	5.32	7.51	0.27***	−0.02***	1			
4 CON	5.85	7.57	0.35***	−0.01	0.3***	1		
5 ST	6.70	8.22	0.33***	−0.05***	0.47***	0.5***	1	
6 SE	5.78	7.83	0.46***	−0.05***	0.28***	0.25***	0.34***	1

*Note:* *p < 0.05; **p < 0.01; ***p < 0.001.

Abbreviations: CON, conservation; NA, negative affect; OTC, openness to change; PA, positive affect; SE, self‐enhancement; ST, self‐transcendence.

^a^ Because the text‐based scores represent the rate of words out of total words used, they are multiplied by 100 for easier interpretations.

**TABLE 6:** Results of the cross‐lagged panel models examining the bi‐directional relationship between affect and Anxiety‐free values (*N* = 14,020, Study 2).

	Openness to change					Self‐transcendence				
	*β*	SE	*p*	95% CI		*β*	SE	*p*	95% CI	
				LL	UL				LL	UL
Autoregressive paths										
Value	0.019	0.009	0.040	0.001	0.037	0.044	0.010	< 0.001	0.024	0.063
PA	0.094	0.011	< 0.001	0.073	0.116	0.088	0.011	< 0.001	0.067	0.110
NA	0.088	0.009	< 0.001	0.070	0.107	0.088	0.009	< 0.001	0.069	0.106
Cross‐lagged paths										
PA_T1_ ➔ Value_T2_	0.033	0.010	0.001	0.014	0.052	0.032	0.010	0.001	0.013	0.050
NA_T1_ ➔ Value_T2_	−0.011	0.009	0.251	−0.029	0.008	−0.015	0.009	0.104	−0.033	0.003
Value_T1_ ➔ PA_T2_	0.033	0.010	0.001	0.014	0.052	0.043	0.010	< 0.001	0.023	0.062
Value_T1_ ➔ NA_T2_	−0.015	0.009	0.105	−0.032	0.003	−0.014	0.009	0.128	−0.033	0.004
NA_T1_ ➔ PA_T2_	−0.016	0.008	0.061	−0.033	0.001	−0.015	0.009	0.089	−0.031	0.002
PA_T1_ ➔ NA_T2_	−0.003	0.010	0.751	−0.023	0.017	−0.002	0.010	0.833	−0.023	0.018
Variance explained (*R* ^*2*^)										
Value_T2_	0.002					0.004				
PA_T2_	0.012					0.013				
NA_T2_	0.008					0.008				

*Note:* Each cross‐lagged panel model was fitted for one higher order value and the two affect types.

Abbreviations: NA, negative affect; PA, positive affect.

**TABLE 7:** Results of the cross‐lagged panel models examining the bi‐directional relationship between affect and anxiety‐based values (*N* = 14,020, Study 2).

	Conservation					Self‐enhancement				
	*β*	SE	*p*	95% CI		*β*	SE	*p*	95% CI	
				LL	UL				LL	UL
Autoregressive paths										
Value	0.052	0.010	< 0.001	0.032	0.072	0.031	0.011	0.004	0.010	0.052
PA	0.089	0.011	< 0.001	0.067	0.110	0.095	0.012	< 0.001	0.072	0.119
NA	0.089	0.009	< 0.001	0.070	0.107	0.088	0.009	< 0.001	0.069	0.106
Cross‐lagged paths										
PA_T1_ ➔ Value_T2_	0.007	0.009	0.482	−0.012	0.025	0.036	0.010	< 0.001	0.016	0.056
NA_T1_ ➔ Value_T2_	−0.005	0.010	0.632	−0.023	0.014	−0.027	0.009	0.003	−0.045	−0.009
Value_T1_ ➔ PA_T2_	0.037	0.011	< 0.001	0.016	0.058	0.017	0.011	0.108	−0.004	0.038
Value_T1_ ➔ NA_T2_	−0.036	0.010	< 0.001	−0.055	−0.016	−0.016	0.010	0.109	−0.036	0.004
NA_T1_ ➔ PA_T2_	−0.016	0.009	0.065	−0.032	0.001	−0.015	0.009	0.084	−0.031	0.002
PA_T1_ ➔ NA_T2_	0.007	0.011	0.494	−0.014	0.028	0.001	0.011	0.963	−0.021	0.022
Variance explained (*R* ^*2*^)										
Value_T2_	0.003					0.004				
PA_T2_	0.012					0.011				
NA_T2_	0.009					0.008				

*Note:* Each cross‐lagged panel model was fitted for one higher order value and the two affect types.

Abbreviations: NA, negative affect; PA, positive affect.

Next, we analyzed the lagged effect of affect at Time 1 on values at Time 2. Results showed that Time 1 positive affect positively predicted Time 2 OTC and ST, indicating that individuals with more positive affect at Time 1 expressed higher levels of OTC and ST at Time 2. Time 2 SE were positively predicted by Time 1 positive affect and negatively predicted by Time 1 negative affect, indicating that individuals with higher positive affect and lower negative affect at Time 1 expressed higher levels of SE. Notably, Time 2 CON were not predicted by any past affective experiences.

The CLPMs also revealed the influence of values on affect over time. Time 1 OTC positively predicted Time 2 positive affect, suggesting that individuals with higher OTC at Time 1 experienced greater positive affect after the city lockdown. Time 1 CON positively predicted Time 2 positive affect and negatively predicted Time 2 negative affect, indicating that individuals with higher CON at Time 1 reported greater happiness and less unhappiness after the lockdown. Time 1 ST positively predicted Time 2 positive affect, suggesting that individuals prioritizing ST experienced greater happiness after the lockdown.

Overall, our findings from real‐time panel data using lexical analysis reflect a temporal relationship between past affect and anxiety‐free values, consistent with the results from Study 1. Additionally, similar to the cross‐sectional results in Study 1, Study 2 observed that individuals who experienced fewer negative feelings or more positive feelings had higher SE, reflecting reward‐seeking motivation (Leikas et al. 2009) or medium‐related effects unique to text‐based scores. Consistent with Study 1, Study 2 found no relationship between CON and past affect, regardless of affect's pleasantness.

In addition, the findings also revealed a reciprocal influence of values on affect over time. Study 2 showed that people prioritizing ST and CON values exhibited better adaptation after the city lockdown than those with lower ST and CON, reflecting the fulfillment of goals toward stability, safety, and caring for others. Consistent with previous findings (e.g., Bojanowska et al. 2021), individuals with higher OTC reported greater positive affect after the lockdown. Further CLPM results of basic values showed that among the three OTC values, only hedonism predicted higher positive affect. This finding could be attributed to hedonistic attitudes and behaviors adopted by individuals prioritizing hedonism (Veenhoven 2003), which likely facilitated better adaptation to the abrupt changes in daily routines during a city lockdown.

## 4 General Discussion

Using cross‐sectional and panel data from self‐reports and lexical analysis, this research explored how past affective experiences might relate to people's value development. Several key findings emerged. First, anxiety‐free values, including ST and OTC, showed close relationships with past positive affective experiences. Second, CON consistently demonstrated no relationship with any affective experiences. Third, the inconsistent relationship between SE and affect across methods and data suggests a more complex relationship between Achievement and affect in terms of regulatory focus. Each finding is discussed in more detail in the following sections.

### 4.1 Anxiety‐Free Values Reinforced by Positive Affect

Consistent with existing theories, our results showed a positive correlation between anxiety‐free values and past positive affect. Specifically, ST demonstrated a consistent and robust positive correlation with positive affect across data and study designs. This positive correlation suggests that living a happy life fosters a tendency to prioritize the greater good over personal needs, aligning with research on the mutual reinforcement between positive affect (e.g., happiness and gratitude) and prosocial behaviors and cooperation (Aknin et al. 2012; Bartlett and DeSteno 2006). Our findings indicate that positive affect promotes ST, which may then motivate cooperative and altruistic behaviors (Sagiv and Schwartz 2022).

Our results also showed a positive correlation between past positive affect and OTC, indicating that happier people tend to prioritize independence, novelty, and personal growth. This result aligns with our theoretical frameworks, suggesting that life satisfaction provides emotional resources for pursuing self‐interest and growth (Schwartz and Sortheix 2018). Fredrickson's (2001) broaden‐and‐build theory argues that positive affect (e.g., joy, interest, pride, and love) broadens thoughts and actions, fostering exploration, creativity, and learning. Supporting evidence indicates that positive affect drives intrinsic motivation (Bloom and Colbert 2011) and curiosity‐driven tasks (Isen and Reeve 2005), which aligns with our finding that positive experiences foster OTC focused on self‐expansion.

### 4.2 Conservation Values Unrelated to Affective Experiences

Our results from both cross‐sectional and panel data, using self‐reports and lexical analysis, showed no link between CON and past affect, contradicting our hypothesis that negative affect would motivate the pursuit of anxiety‐based values.

The consistent but non‐significant results may suggest alternative pathways for value development beyond internal psychological processes. Researchers have argued that values can be directly transmitted through socialization and persuasion (Bardi and Goodwin 2011), acquired through interactions with media, parents, education, and cultural contexts (Chatard and Selimbegovic 2007; Grusec and Goodnow 1994). Ahn and Reeve (2021) specifically examined these distinct pathways of value development among adolescents over a year, finding that intrinsic values (similar to anxiety‐free values; Schwartz 2016a) develop through innate psychological experiences like need fulfillment or frustration, whereas extrinsic values (similar to anxiety‐based values) are often directly transmitted from external sources like mothers.

The direct transmission of CON through socialization is particularly relevant for mainland Chinese participants in our research. Congruent with CON, maintaining and facilitating the harmony and cohesiveness in the groups is highly appreciated in group‐oriented Chinese cultures (Leung 2010). Rather than being primarily driven by negative affect, individuals in Chinese cultures may endorse and internalize CON values through socialization processes, viewing them as social norms and integral aspects of self (Chen 2010, 38–39; Liu et al. 2010, 580).

Building on prior research, the non‐significant effect of affect on CON observed in our study may reflect direct socialization rather than internal psychological processes. Further exploration is needed to better understand these divergent mechanisms of value development, particularly within Chinese cultures.

### 4.3 Self‐Enhancement Values Related to Both Positive and Negative Affect

Contrary to our initial hypotheses, our results showed a positive association between SE and past affective experience characterized by lower negative affect or higher positive affect. This unexpected finding may relate to the complex anxiety‐related motivations underlying achievement, which fall under the broader SE category.

While SE is generally argued to stem from the need to control anxiety (Schwartz 2016a) and was thus hypothesized to correlate with negative affect, achievement serves more complex motivational goals concerning their relationship with anxiety. Schwartz (2016a) identified achievement as addressing both anxiety‐based needs (e.g., meeting social standards to alleviate anxiety) and anxiety‐free needs (e.g., affirming competence and expressing the self). Relatedly, Chinese students' motivation for academic achievement was found to be related to both social obligations and self‐fulfillment (Hau and Ho 2010, 189–191).

Previous research typically views Achievement as promotion‐focused, linking it to positive affective experiences (e.g., Leikas et al. 2009; Sagiv and Schwartz 2000). Our CLPM result of 10 basic values (see Table S6) confirmed this link—Achievement at Time 2 was positively predicted by past positive affect and negatively predicted by past negative affect. These exploratory findings, consistent with existing evidence, help explain the unexpected positive association between SE and positive affect in our main results.

A notable discrepancy between self‐report and text‐based scores is that while positive associations were consistently found between positive affect and SE for the text‐based scores (see Table 4 and Table 7), this pattern was absent in self‐reports. In fact, self‐report SE showed a positive association with negative affect and exhibited a negative, albeit non‐significant, association with positive affect (see Table 4, upper section). This discrepancy likely reflects medium‐specific effects unique to the lexical approach in the following aspects.

First, text‐based expressions of values may differ from self‐reports due to the spontaneous nature of language use. In everyday communication, discussions about achievement are often framed positively, tied to past successes or collaborative experiences. To explore this pattern in our data, we used a feature co‐occurrence matrix (FCM)—a lexical analysis method that examines co‐occurrence patterns of words in texts. Our Study 1's text data showed that “成就” (achievement) co‐occurred with positive terms like “开心” (happy), “快乐” (joy), and “温暖” (warmth), linking achievement to positive affect in everyday language. In contrast, self‐report measures may miss this pattern, as participants evaluate each concept separately per explicit instructions.

Second, the positive associations of SE with positive affect may originate from language expressivity in communication influenced by social desirability. Individuals often use positive language in public contexts (e.g., interviews and social media; Bergen and Labonté 2020; Chen and Yik 2022), and those with stronger social desirability tend to report more positive affect (Soubelet and Salthouse 2011), potentially influencing value expression. For example, content related to SE (e.g., Power, reflecting self‐promotion) and ST (e.g., Benevolence, reflecting care for others) may serve to project a positive self‐image, meeting the needs of individuals with stronger social desirability. Supporting this, text‐based Power and Benevolence were positively correlated with MRAT scores (see Table 1)—a social desirability proxy (Borg and Bardi 2016)—suggesting that shared desirability biases underpin the parallel affect–value relationships observed in text‐based SE and ST analyses.

### 4.4 Low Convergence Between Self‐Report and Text‐Based Conservation Values

In this research, we developed the Chinese Personal Values Dictionary (CPVD) to assess personal values in texts. Our validation results showed that the scores elicited using CPVD exhibited similarities with self‐report values, with correlations comparable to previously developed value dictionaries (e.g., Fischer and Karl 2023; Ponizovskiy et al. 2020). CLPM's significant autoregressive effects also demonstrated CPVD's temporal stability.

A notable finding was the low convergence between self‐report CON and text‐based CON. To explore this discrepancy, we generated a word cloud (see supplementary Figure S1) showing the most frequent nouns and adjectives used by participants high in self‐report CON, with larger font size indicating higher usage frequency. The resulting word cloud revealed two distinct lists of words related to traditional Chinese values and principles. One set reflected the Confucian principle of “Li” (rites, decorum, and norms that promote interpersonal harmony and mutual responsibility; Chen and Starosta 1997). For example, words like “尊重 (respect),” “诚实 (honesty),” and “真诚 (genuineness)” were frequently used by Chinese participants high in CON, likely due to their roles in guiding proper conduct in Chinese culture. Another set of words reflected close familial relationships, particularly concerning “孝” (filial piety) or “家” (family, parenting; Kulich and Zhang 2010). Words like “父母” (parents), “孩子” (children), “家人” (family members), and “家庭” (family) appeared prominently in the word cloud, suggesting that CON may be strongly rooted in obligations and priorities related to the family unit.

The low convergence between self‐report and text‐based CON likely reflects cultural differences. The unique content and expression of CON in Chinese contexts likely underlie the low convergence between self‐report and text‐based measures. For example, the CPVD, built based on the Western‐based PVD—which lacks terms reflecting Confucian, Buddhist, or Taoist influences in Chinese culture—may inadequately represent the indigenous concepts of tradition and conformity. Consequently, the results for text‐based CON should be interpreted with caution. Adding terms tied to traditional morality and social principles could strengthen Chinese value lexicons' ability to measure CON values.

### 4.5 Theoretical and Methodological Contributions

The present research makes important theoretical and methodological contributions. Theoretically, we drew upon the Theory of Basic Human Values (Schwartz 1992), the motivational perspective of affect (Lang and Bradley 2010), and Higgins (1997) self‐regulation systems to examine affect's potential causal influence on value development. Using both cross‐sectional and panel data derived from self‐report measures and lexical approaches, our findings illuminate how past affective experiences may shape the personal values people pursue. Overall, our results suggest complex mechanisms underlying value development, extending beyond the simple dichotomy of anxiety‐based versus anxiety‐free values. The present research emphasizes the importance of examining affective experiences, in addition to other socialization factors, to fully capture the dynamic process through which personal values emerge and evolve over time.

The present research also offers two significant methodological contributions. First, we developed a lexical analysis tool, the Chinese Personal Values Dictionary (CPVD), for assessing personal values in Chinese texts. Our validation results indicated that CPVD‐predicted values exhibited similarities with self‐report values, comparable to those reported for other value dictionaries.

Second, we complemented traditional self‐reports with lexical analysis—an objective, efficient, and resource‐saving method for assessing values that enables large‐scale, real‐time analysis using diverse texts (Pennebaker et al. 2003). Importantly, the convergent findings across our lexical analysis and self‐reports have cross‐validated and expanded our understanding of the complex relationship between values and affect through both objective and subjective measures.

### 4.6 Limitations and Future Directions

The present research has several limitations. First, using naturally observed data limits causal inferences about past affect's influence on values. Nevertheless, Study 2's panel data analysis controlled for autoregressive effects, revealing how earlier affect may influence later values—providing preliminary evidence for potential causality (Selig and Little 2012), though future experimental studies are needed to test the causal influence of affect on values.

Second, we note that the effect sizes of text‐based scores are sometimes small. This is a common limitation in lexical analysis due to various sources of noise, such as contextual information and idiomatic expressions (Iliev et al. 2015). While small effect sizes may raise concerns about overemphasizing statistical significance, particularly in large datasets, they remain meaningful in psychological research (Götz et al. 2022; Matz et al. 2017). For instance, small effects enable a nuanced exploration of complex psychological phenomena that larger effects might obscure. Moreover, even minor changes, when aggregated across large populations, can result in significant societal outcomes. Our self‐report and text‐based measures yielded convergent results for anxiety‐free values, bolstering confidence in the findings. Future studies could further validate these findings using panel data or experimental designs.

Third, we used a word‐counting approach for lexical analysis, which has inherent limitations compared to more advanced techniques. While providing a basic quantitative measure of language use, word‐counting methods are less effective at capturing semantic and contextual information. For example, “家人” (translated from either “family” or “relatives” from the original PVD) could align with both Benevolence and Tradition values, and was rated as representing both values similarly by the 10 raters in Study 1,^11^ highlighting the overlapped value concepts within the same word. We also conducted an FCM analysis to explore the contextual effects. The results revealed contextual differences: Individuals high in ST associated “家人” with “诚实” (honesty) and “信任” (trust), whereas individuals high in CON associated “家人” with “感受” (feelings) and “人生” (life). Although the pattern is not yet definitive, these results suggest that contextual information in language use may carry unique value‐related concerns.

As noted, the word‐counting approach is still commonly used as a starting point in psychological research. Future studies could explore the use of more sophisticated lexical analysis tools, such as the Fill‐Mask Association Test (FMAT; Bao 2024), the Masked Language Model (MLM; Starovolsky‐Shitrit et al. 2025), and the Large Language Models (LLM; Ye et al. 2024), which can better account for the contextual usage of words.

## 5 Conclusion

Across both cross‐sectional and panel data using self‐report measures and lexical approaches, our findings suggest that affective experiences play a significant role in shaping the development of personal values over time. Importantly, this work highlights the potential plasticity of affective experiences, which may serve as a more malleable pathway for interventions aimed at facilitating the development of personal values.

The lexical analysis approach applied in this research demonstrates the valuable utility of leveraging real‐world language data to investigate psychological constructs. A lexical approach allows for rapid, efficient, and potentially more replicable investigations compared to traditional survey‐based studies, providing innovative insights into psychological questions. This method shows promise for examining psychological issues using various text resources, such as social media data, thereby expanding its application potential.

## Author Contributions

**Xi Chen:** conceptualization, data curation, formal analysis, writing – original draft, review, and editing. **Shengquan Ye:** conceptualization, supervision, writing – review and editing. All authors have read and approved the final manuscript.

## Conflicts of Interest

The authors declare no conflicts of interest.

## Supporting information


**Data S1:** jopy70022‐sup‐0001‐Supinfo.docx.

## Endnotes

^1^
Achievement, which fall under the broader SE category, can serve as both anxiety‐based (meeting the social standards to relieve anxiety) or anxiety‐free values (affirm competence and express the self; Schwartz 2016a).

^2^
We focused on Chinese language because previous studies mainly recruited participants in non‐Asian cultures (e.g., New Zealand in Fischer and Karl 2023; Isreal, West Germany, and East Germany in Sagiv and Schwartz 2000). We know little about how affect might influence values in Asian cultures (e.g., Chinese culture).

^3^
For example, GPT‐4 translated “dash” (categorized in Self‐direction) into terms like “破折号” (punctuation mark) and “冲刺” (short burst of speed), which were unrelated to Self‐direction. The human translator removed these translations and added more appropriate translations, including “冲劲” (drive or momentum to solving challenges) and “锐气” (sharpness in spirit).

^4^
HIT IR‐Lab Tongyici Cilin (Extended), developed by the Research Center for Social Computing and Information Retrieval at Harbin Institute of Technology (HIT‐SCIR; see https://www.ltp‐cloud.com/), is widely used for Chinese text processing (Liang et al. 2013).

^5^
Both CPVD versions (1033 and 8906 words) are available on OSF (https://osf.io/24pe8/files/osfstorage).

^6^
The negation word list, sourced from https://github.com/DavionWu2018/Sentiment_dictionary, comprises 71 terms, including “no” or “not” (e.g., “不是”, “否”) and verbs with negative connotations (e.g., “反对” [oppose]).

^7^
For exploratory purposes, we also tested the effect of recalled arousal on values (see Table S2).

^8^

https://github.com/nghuyong/weibo‐cov.

^9^
Sina Weibo is a Chinese social media platform where users share short messages, images, and videos while interacting via comments and shares. With over 500 million monthly active users (Weibo 2020), it covers users of different ages and genders from different regions of China.

^10^
The panel data (previously used in Chen and Yik 2022) included the following: (1) posts from January 9 to February 6, 2020 (spanning 2 weeks before to 2 weeks after Wuhan lockdown); (2) posts from users with GPS coordinates; (3) excluded users with just one post during this period.

^11^
A paired t‐test, albeit in small sample size, yield *t* (9) = −1.25, *p* = 0.24.
