# Supplementary material for: From Affect to Values: A Lexical Approach
Source: J Pers. 2025 Sep 17;94(4):563–83. doi: 10.1111/jopy.70022 (PMC13359312; doi:10.1111/jopy.70022)
Supplement: Supplementary file 1 — Data S1: jopy70022‐sup‐0001‐Supinfo.docx. [file JOPY-94-563-s001.docx]

**Figure S1**

*Word Cloud Demonstrating Frequently Used Words by Participants with High Levels of Self-report Conservation Values*


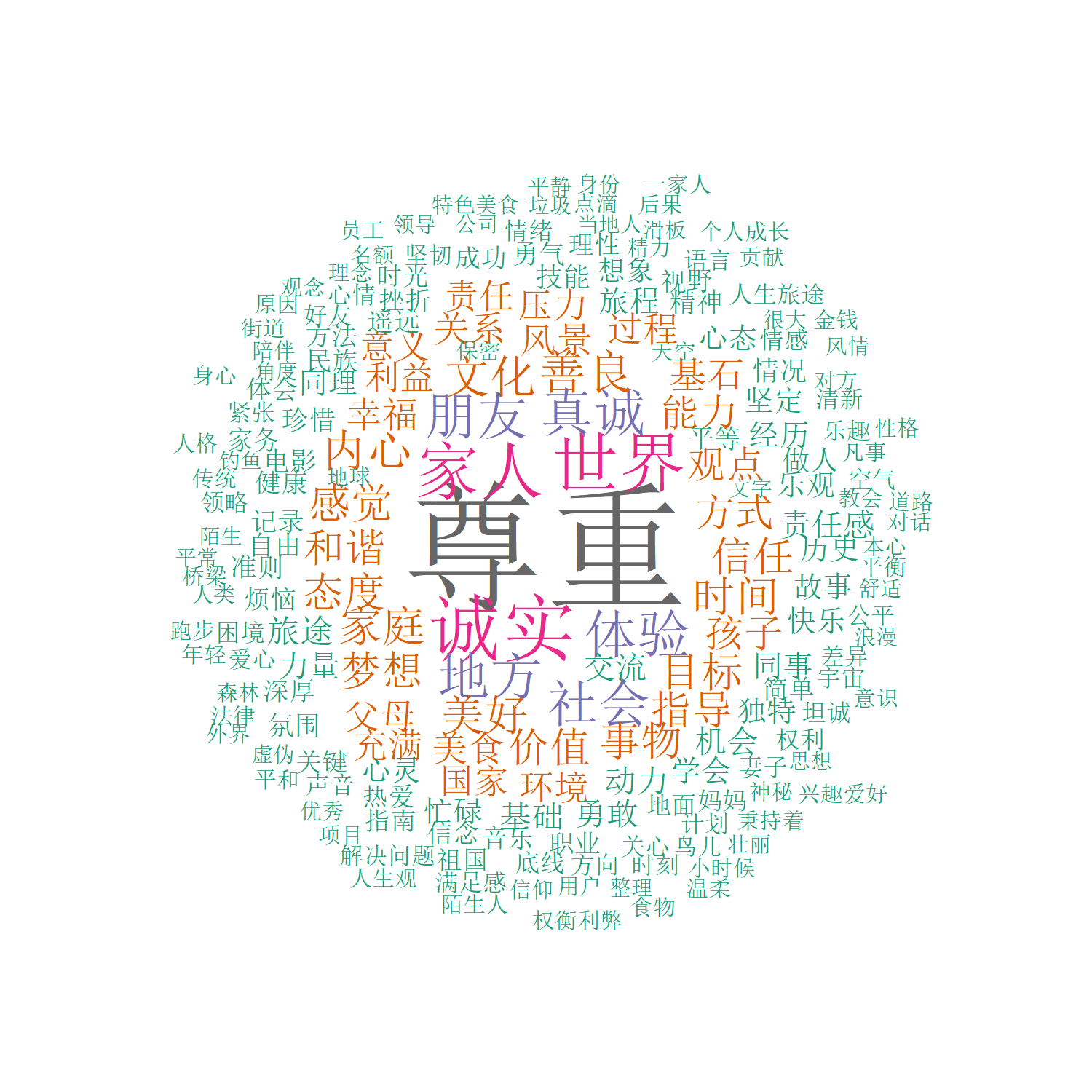


**Table S1**

*Results of Multivariate Models Examining the Effect of Recalled Affect on 10 Basic Values (N=230, Study 2)*

|  | *β* | *SE* | *P* | 95% CI | | *R^2^* |
| --- | --- | --- | --- | --- | --- | --- |
|  |  |  |  | *LL* | *UL* |  |
| Self-report |  |  |  |  |  |  |
| Security |  |  |  |  |  | 0.056 |
| Positive affect | **-0.262** | **0.074** | **<.001** | -0.407 | -0.117 |  |
| Negative affect | -0.054 | 0.077 | .477 | -0.205 | 0.096 |  |
| Conformity |  |  |  |  |  | 0.022 |
| Positive affect | 0.098 | 0.078 | .209 | -0.055 | 0.250 |  |
| Negative affect | -0.071 | 0.078 | .359 | -0.224 | 0.081 |  |
| Tradition |  |  |  |  |  | 0.033 |
| Positive affect | **0.199** | **0.076** | **.009** | 0.050 | 0.348 |  |
| Negative affect | 0.036 | 0.078 | .645 | -0.116 | 0.188 |  |
| Benevolence |  |  |  |  |  | 0.196 |
| Positive affect | **0.411** | **0.065** | **<.001** | 0.284 | 0.538 |  |
| Negative affect | -0.053 | 0.071 | .450 | -0.192 | 0.085 |  |
| Universalism |  |  |  |  |  | 0.220 |
| Positive affect | **0.376** | **0.065** | **.000** | 0.249 | 0.503 |  |
| Negative affect | **-0.141** | **0.069** | **.041** | -0.276 | -0.006 |  |
| Self-direction |  |  |  |  |  | 0.202 |
| Positive affect | **0.369** | **0.066** | **<.001** | 0.240 | 0.497 |  |
| Negative affect | -0.124 | 0.070 | 0.076 | -0.261 | 0.013 |  |
| Stimulation |  |  |  |  |  | 0.202 |
| Positive affect | **0.442** | **0.063** | **<.001** | 0.317 | 0.566 |  |
| Negative affect | -0.014 | 0.071 | 0.838 | -0.153 | 0.124 |  |
| Hedonism |  |  |  |  |  | 0.026 |
| Positive affect | **0.184** | **0.077** | **.016** | 0.034 | 0.334 |  |
| Negative affect | 0.055 | 0.078 | .479 | -0.097 | 0.208 |  |
| Achievement |  |  |  |  |  | 0.003 |
| Positive affect | -0.039 | 0.079 | .619 | -0.194 | 0.115 |  |
| Negative affect | 0.026 | 0.079 | .741 | -0.128 | 0.180 |  |
| Power |  |  |  |  |  | 0.094 |
| Positive affect | -0.079 | 0.075 | .293 | -0.226 | 0.068 |  |
| Negative affect | **0.257** | **0.073** | **<.001** | 0.115 | 0.399 |  |
| Text-based |  |  |  |  |  |  |
| Security |  |  |  |  |  | 0.041 |
| Positive affect | **0.141** | **0.068** | **.038** | 0.008 | 0.275 |  |
| Negative affect | -0.103 | 0.069 | .132 | -0.238 | 0.031 |  |
| Conformity |  |  |  |  |  | 0.005 |
| Positive affect | 0.068 | 0.070 | .330 | -0.069 | 0.206 |  |
| Negative affect | 0.056 | 0.070 | .424 | -0.082 | 0.194 |  |
| Tradition |  |  |  |  |  | 0.010 |
| Positive affect | -0.076 | 0.070 | .275 | -0.213 | 0.061 |  |
| Negative affect | -0.099 | 0.070 | .156 | -0.236 | 0.038 |  |
| Benevolence |  |  |  |  |  | 0.092 |
| Positive affect | **0.250** | **0.065** | **<.001** | 0.123 | 0.376 |  |
| Negative affect | -0.106 | 0.067 | .113 | -0.237 | 0.025 |  |
| Universalism |  |  |  |  |  | 0.026 |
| Positive affect | 0.124 | 0.069 | .071 | -0.011 | 0.260 |  |
| Negative affect | -0.067 | 0.069 | .333 | -0.203 | 0.069 |  |
| Self-direction |  |  |  |  |  | 0.035 |
| Positive affect | -0.134 | 0.069 | .050 | -0.268 | 0.000 |  |
| Negative affect | **-0.186** | **0.068** | **.006** | -0.318 | -0.053 |  |
| Stimulation |  |  |  |  |  | 0.055 |
| Positive affect | **0.237** | **0.066** | **<.001** | 0.107 | 0.366 |  |
| Negative affect | 0.006 | 0.069 | .925 | -0.128 | 0.141 |  |
| Hedonism |  |  |  |  |  | 0.003 |
| Positive affect | 0.059 | 0.070 | .404 | -0.079 | 0.197 |  |
| Negative affect | 0.022 | 0.070 | .759 | -0.116 | 0.160 |  |
| Achievement |  |  |  |  |  | 0.051 |
| Positive affect | **0.172** | **0.067** | **.011** | 0.039 | 0.304 |  |
| Negative affect | -0.099 | 0.068 | .149 | -0.233 | 0.035 |  |
| Power |  |  |  |  |  | 0.059 |
| Positive affect | **0.244** | **0.066** | **<.001** | 0.115 | 0.373 |  |
| Negative affect | **0.168** | **0.067** | **.012** | 0.036 | 0.300 |  |

*Note*. Significant results are bolded. Raw scores are used for the self-report variables.

**Table S2**

*Results of Multivariate Models Examining the Effect of Recalled Arousal on Values (N=230, Study 2 Phase 3)*

|  | *β* | *SE* | *P* | 95% CI | | *R^2^* |
| --- | --- | --- | --- | --- | --- | --- |
|  |  |  |  | *LL* | *UL* |  |
| Self-report^a^ |  |  |  |  |  |  |
| Openness to Change |  |  |  |  |  | 0.282 |
| High Arousal Positive | **0.529** | **0.058** | **<.001** | 0.416 | 0.642 |  |
| Low Arousal Positive | -0.039 | 0.066 | .549 | -0.168 | 0.089 |  |
| High Arousal Negative | **-0.143** | **0.067** | **.034** | -0.275 | -0.011 |  |
| Low Arousal Negative | 0.061 | 0.074 | .410 | -0.084 | 0.206 |  |
| Conservation |  |  |  |  |  | 0.039 |
| High Arousal Positive | -0.135 | 0.077 | .079 | -0.286 | 0.016 |  |
| Low Arousal Positive | **0.212** | **0.074** | **.004** | 0.066 | 0.357 |  |
| High Arousal Negative | -0.002 | 0.079 | .982 | -0.156 | 0.153 |  |
| Low Arousal Negative | -0.005 | 0.086 | .954 | -0.173 | 0.163 |  |
| Self-transcendence |  |  |  |  |  | 0.273 |
| High Arousal Positive | **0.238** | **0.066** | **<.001** | 0.109 | 0.367 |  |
| Low Arousal Positive | **0.233** | **0.064** | **<.001** | 0.107 | 0.359 |  |
| High Arousal Negative | 0.017 | 0.068 | .806 | -0.117 | 0.151 |  |
| Low Arousal Negative | **-0.197** | **0.073** | **.007** | -0.341 | -0.054 |  |
| Self-enhancement |  |  |  |  |  | 0.054 |
| High Arousal Positive | 0.074 | 0.077 | .338 | -0.077 | 0.224 |  |
| Low Arousal Positive | -0.127 | 0.075 | .089 | -0.274 | 0.019 |  |
| High Arousal Negative | 0.046 | 0.078 | .552 | -0.107 | 0.199 |  |
| Low Arousal Negative | 0.152 | 0.084 | .071 | -0.013 | 0.316 |  |
| Text-based^b^ |  |  |  |  |  |  |
| Openness to Change |  |  |  |  |  | 0.015 |
| High Arousal Positive | 0.026 | 0.069 | .702 | -0.109 | 0.162 |  |
| Low Arousal Positive | 0.007 | 0.073 | .923 | -0.136 | 0.150 |  |
| High Arousal Negative | -0.016 | 0.072 | .830 | -0.158 | 0.126 |  |
| Low Arousal Negative | -0.106 | 0.071 | .136 | -0.245 | 0.033 |  |
| Conservation |  |  |  |  |  | 0.017 |
| High Arousal Positive | -0.009 | 0.069 | .897 | -0.144 | 0.126 |  |
| Low Arousal Positive | 0.070 | 0.073 | .337 | -0.073 | 0.212 |  |
| High Arousal Negative | -0.057 | 0.072 | .426 | -0.199 | 0.084 |  |
| Low Arousal Negative | -0.054 | 0.071 | .449 | -0.193 | 0.086 |  |
| Self-transcendence |  |  |  |  |  | 0.071 |
| High Arousal Positive | 0.010 | 0.067 | .882 | -0.122 | 0.142 |  |
| Low Arousal Positive | **0.221** | **0.069** | **.001** | 0.086 | 0.355 |  |
| High Arousal Negative | -0.007 | 0.07 | .917 | -0.145 | 0.131 |  |
| Low Arousal Negative | -0.093 | 0.069 | .176 | -0.229 | 0.042 |  |
| Self-enhancement |  |  |  |  |  | 0.064 |
| High Arousal Positive | -0.024 | 0.067 | .717 | -0.157 | 0.108 |  |
| Low Arousal Positive | **0.262** | **0.068** | **.000** | 0.129 | 0.396 |  |
| High Arousal Negative | 0.026 | 0.071 | .712 | -0.112 | 0.165 |  |
| Low Arousal Negative | -0.012 | 0.070 | .859 | -0.149 | 0.124 |  |

*Note*. Significant results are bolded. Raw scores are used for self-report variables.

^a^ Self-report affect scores were computed for high-arousal positive (HAP), low-arousal positive (LAP), high-arousal negative (HAN), and low-arousal negative (LAN) affect using the Affect Valuation Index (Tsai et al., 2006).

^b^ To calculate text-based affect with varied level of arousal, the affect words were first categorized into HAP, LAP, HAN, and LAN based on their valence and arousal scores in the lexicon. The percentage of word frequency of each affect category over total word were used to represent the affect scores.

**Table S3**

*Results of Cross-Lagged Panel Models Examining the Effect of Recalled Affect on Basic Values of Openness to Change (N=14,020, Study 2)*

|  | Self-direction | | | | | Stimulation | | | | | Hedonism | | | | |
| --- | --- | --- | --- | --- | --- | --- | --- | --- | --- | --- | --- | --- | --- | --- | --- |
|  | *β* | *SE* | *P* | 95% CI | | *β* | *SE* | *P* | 95% CI | | *β* | *SE* | *P* | 95% CI | |
|  |  |  |  | *LL* | *UL* |  |  |  | *LL* | *UL* |  |  |  | *LL* | *UL* |
| Autoregressive Paths |  |  |  |  |  |  |  |  |  |  |  |  |  |  |  |
| Value | 0.014 | 0.011 | .214 | -0.008 | 0.035 | 0.047 | 0.011 | <.001 | 0.025 | 0.069 | 0.025 | 0.011 | .024 | 0.003 | 0.047 |
| PA | 0.103 | 0.012 | <.001 | 0.079 | 0.127 | 0.104 | 0.012 | <.001 | 0.081 | 0.128 | 0.101 | 0.012 | <.001 | 0.078 | 0.125 |
| NA | 0.092 | 0.011 | <.001 | 0.070 | 0.114 | 0.092 | 0.011 | <.001 | 0.069 | 0.114 | 0.089 | 0.011 | <.001 | 0.067 | 0.112 |
| Cross-lagged Paths |  |  |  |  |  |  |  |  |  |  |  |  |  |  |  |
| PA_T1_ 🡪 Value_T2_ | 0.012 | 0.013 | .361 | -0.014 | 0.038 | -0.021 | 0.009 | .024 | -0.039 | -0.003 | 0.023 | 0.011 | .044 | 0.001 | 0.045 |
| NA_T1_ 🡪 Value_T2_ | 0.018 | 0.011 | .090 | -0.003 | 0.040 | -0.013 | 0.010 | .191 | -0.032 | 0.006 | -0.016 | 0.012 | .210 | -0.040 | 0.009 |
| Value_T1_ 🡪 PA_T2_ | -0.014 | 0.010 | .187 | -0.034 | 0.007 | 0.004 | 0.010 | .676 | -0.015 | 0.023 | 0.040 | 0.011 | <.001 | 0.018 | 0.062 |
| Value_T1_ 🡪 NA_T2_ | 0.004 | 0.011 | .719 | -0.018 | 0.026 | -0.011 | 0.010 | .269 | -0.032 | 0.009 | -0.030 | 0.011 | .009 | -0.052 | -0.007 |
| NA_T1_ 🡪 PA_T2_ | -0.030 | 0.010 | .004 | -0.051 | -0.010 | -0.030 | 0.010 | .004 | -0.051 | -0.010 | -0.027 | 0.010 | .011 | -0.047 | -0.006 |
| PA_T1_ 🡪 NA_T2_ | -0.028 | 0.011 | .011 | -0.049 | -0.006 | -0.028 | 0.011 | .010 | -0.049 | -0.007 | -0.026 | 0.011 | .017 | -0.047 | -0.005 |
| Variance Explained (*R^2^*) |  |  |  |  |  |  |  |  |  |  |  |  |  |  |  |
| Value_T2_ | 0.001 |  |  |  |  | 0.003 |  |  |  |  | 0.002 |  |  |  |  |
| PA_T2_ | 0.014 |  |  |  |  | 0.013 |  |  |  |  | 0.015 |  |  |  |  |
| NA_T2_ | 0.011 |  |  |  |  | 0.011 |  |  |  |  | 0.011 |  |  |  |  |

*Note*. Each Cross-lagged panel model was fitted for one higher order value and the two affect types. PA: Positive Affect; NA: Negative Affect.

**Table S4**

*Results of Cross-Lagged Panel Models Examining the Effect of Recalled Affect on Basic Values of Conservation (N=14,020, Study 2)*

|  | Security | | | | | Conformity | | | | | Tradition | | | | |
| --- | --- | --- | --- | --- | --- | --- | --- | --- | --- | --- | --- | --- | --- | --- | --- |
|  | *β* | *SE* | *P* | 95% CI | | *β* | *SE* | *P* | 95% CI | | *β* | *SE* | *P* | 95% CI | |
|  |  |  |  | *LL* | *UL* |  |  |  | *LL* | *UL* |  |  |  | *LL* | *UL* |
| Autoregressive Paths |  |  |  |  |  |  |  |  |  |  |  |  |  |  |  |
| Value | 0.047 | 0.011 | <.001 | 0.025 | 0.068 | 0.040 | 0.017 | 0.021 | 0.006 | 0.075 | 0.010 | 0.010 | 0.331 | -0.010 | 0.030 |
| PA | 0.100 | 0.012 | <.001 | 0.076 | 0.124 | 0.104 | 0.012 | <.001 | 0.081 | 0.128 | 0.104 | 0.012 | <.001 | 0.080 | 0.128 |
| NA | 0.087 | 0.011 | <.001 | 0.065 | 0.109 | 0.091 | 0.011 | <.001 | 0.069 | 0.114 | 0.093 | 0.011 | <.001 | 0.070 | 0.115 |
| Cross-lagged Paths |  |  |  |  |  |  |  |  |  |  |  |  |  |  |  |
| PA_T1_ 🡪 Value_T2_ | -0.016 | 0.011 | .153 | -0.038 | 0.006 | 0.001 | 0.015 | .956 | -0.028 | 0.030 | -0.023 | 0.010 | .023 | -0.043 | -0.003 |
| NA_T1_ 🡪 Value_T2_ | -0.010 | 0.011 | .367 | -0.031 | 0.012 | -0.001 | 0.011 | .955 | -0.023 | 0.021 | -0.011 | 0.010 | .307 | -0.031 | 0.010 |
| Value_T1_ 🡪 PA_T2_ | 0.032 | 0.011 | .005 | 0.010 | 0.054 | -0.002 | 0.011 | .891 | -0.024 | 0.021 | -0.006 | 0.009 | .494 | -0.025 | 0.012 |
| Value_T1_ 🡪 NA_T2_ | -0.040 | 0.011 | <.001 | -0.061 | -0.019 | -0.014 | 0.011 | .183 | -0.035 | 0.007 | -0.005 | 0.010 | .594 | -0.025 | 0.014 |
| NA_T1_ 🡪 PA_T2_ | -0.026 | 0.011 | .012 | -0.047 | -0.006 | -0.030 | 0.010 | .004 | -0.051 | -0.010 | -0.030 | 0.010 | .004 | -0.050 | -0.010 |
| PA_T1_ 🡪 NA_T2_ | -0.022 | 0.011 | .040 | -0.044 | -0.001 | -0.029 | 0.011 | .008 | -0.050 | -0.007 | -0.029 | 0.011 | .008 | -0.050 | -0.007 |
| Variance Explained (*R^2^*) |  |  |  |  |  |  |  |  |  |  |  |  |  |  |  |
| Value_T2_ | 0.002 |  |  |  |  | 0.002 |  |  |  |  | 0.001 |  |  |  |  |
| PA_T2_ | 0.014 |  |  |  |  | 0.013 |  |  |  |  | 0.013 |  |  |  |  |
| NA_T2_ | 0.012 |  |  |  |  | 0.011 |  |  |  |  | 0.011 |  |  |  |  |

*Note*. Each Cross-lagged panel model was fitted for one higher order value and the two affect types. PA: Positive Affect; NA: Negative Affect.

**Table S5**

*Results of Cross-Lagged Panel Models Examining the Effect of Recalled Affect on Basic Values of Self-transcendence (N=14,020, Study 2)*

|  | Benevolence | | | | | | | | | | Universalism | | | | | | | | |
| --- | --- | --- | --- | --- | --- | --- | --- | --- | --- | --- | --- | --- | --- | --- | --- | --- | --- | --- | --- |
|  | *β* | | *SE* | | *P* | | 95% CI | | | | *β* | | *SE* | | *P* | | 95% CI | | |
|  |  | |  | |  | | *LL* | | *UL* | |  | |  | |  | | *LL* | | *UL* |
| Autoregressive Paths |  | |  | |  | |  | |  | |  | |  | |  | |  | |  |
| Value | 0.018 | | 0.012 | | .136 | | -0.006 | | 0.041 | | 0.049 | | 0.011 | | <.001 | | 0.027 | | 0.071 |
| PA | 0.104 | | 0.012 | | <.001 | | 0.081 | | 0.128 | | 0.099 | | 0.012 | | <.001 | | 0.076 | | 0.123 |
| NA | 0.092 | | 0.011 | | <.001 | | 0.070 | | 0.114 | | 0.084 | | 0.011 | | <.001 | | 0.061 | | 0.106 |
| Cross-lagged Paths |  |  | |  | |  | |  | |  | |  | |  | |  | |  | |
| PA_T1_ 🡪 Value_T2_ | 0.006 | | 0.012 | | .590 | | -0.017 | | 0.029 | | 0.025 | | 0.012 | | .041 | | 0.001 | | 0.050 |
| NA_T1_ 🡪 Value_T2_ | 0.005 | | 0.011 | | .628 | | -0.017 | | 0.027 | | -0.021 | | 0.011 | | .044 | | -0.042 | | -0.001 |
| Value_T1_ 🡪 PA_T2_ | 0.002 | | 0.010 | | .866 | | -0.018 | | 0.021 | | 0.053 | | 0.012 | | <.001 | | 0.030 | | 0.076 |
| Value_T1_ 🡪 NA_T2_ | -0.005 | | 0.010 | | .609 | | -0.026 | | 0.015 | | -0.048 | | 0.011 | | <.001 | | -0.070 | | -0.026 |
| NA_T1_ 🡪 PA_T2_ | -0.030 | | 0.010 | | .004 | | -0.051 | | -0.010 | | -0.021 | | 0.011 | | .046 | | -0.042 | | 0.000 |
| PA_T1_ 🡪 NA_T2_ | -0.029 | | 0.011 | | .009 | | -0.050 | | -0.007 | | -0.024 | | 0.011 | | .030 | | -0.045 | | -0.002 |
| Variance Explained (*R^2^*) |  |  | |  | |  | |  | |  | |  | |  | |  | |  | |
| Value_T2_ | 0.000 | |  | |  | |  | |  | | 0.004 | |  | |  | |  | |  |
| PA_T2_ | 0.013 | |  | |  | |  | |  | | 0.016 | |  | |  | |  | |  |
| NA_T2_ | 0.011 | |  | |  | |  | |  | | 0.013 | |  | |  | |  | |  |

*Note*. Each Cross-lagged panel model was fitted for one higher order value and the two affect types. PA: Positive Affect; NA: Negative Affect.

**Table S6**

*Results of Cross-Lagged Panel Models Examining the Effect of Recalled Affect on Basic Values of Self-enhancement (N=14,020, Study 2)*

|  | Achievement | | | | | | | | | | Power | | | | | | | | |
| --- | --- | --- | --- | --- | --- | --- | --- | --- | --- | --- | --- | --- | --- | --- | --- | --- | --- | --- | --- |
|  | *β* | | *SE* | | *P* | | 95% CI | | | | *β* | | *SE* | | *P* | | 95% CI | | |
|  |  | |  | |  | | *LL* | | *UL* | |  | |  | |  | | *LL* | | *UL* |
| Autoregressive Paths |  | |  | |  | |  | |  | |  | |  | |  | |  | |  |
| Value | 0.013 | | 0.012 | | .278 | | -0.011 | | 0.037 | | 0.019 | | 0.010 | | .046 | | 0.000 | | 0.038 |
| PA | 0.104 | | 0.012 | | <.001 | | 0.080 | | 0.128 | | 0.103 | | 0.012 | | <.001 | | 0.080 | | 0.127 |
| NA | 0.091 | | 0.011 | | <.001 | | 0.068 | | 0.113 | | 0.092 | | 0.011 | | <.001 | | 0.070 | | 0.114 |
| Cross-lagged Paths |  |  | |  | |  | |  | |  | |  | |  | |  | |  | |
| PA_T1_ 🡪 Value_T2_ | 0.025 | | 0.011 | | .022 | | 0.004 | | 0.047 | | -0.021 | | 0.010 | | .027 | | -0.040 | | -0.002 |
| NA_T1_ 🡪 Value_T2_ | -0.037 | | 0.011 | | <.001 | | -0.058 | | -0.016 | | -0.007 | | 0.011 | | .523 | | -0.030 | | 0.015 |
| Value_T1_ 🡪 PA_T2_ | 0.002 | | 0.011 | | .886 | | -0.020 | | 0.023 | | -0.016 | | 0.010 | | .092 | | -0.035 | | 0.003 |
| Value_T1_ 🡪 NA_T2_ | -0.018 | | 0.011 | | .095 | | -0.040 | | 0.003 | | -0.007 | | 0.011 | | .520 | | -0.028 | | 0.014 |
| NA_T1_ 🡪 PA_T2_ | -0.030 | | 0.010 | | .004 | | -0.051 | | -0.010 | | -0.031 | | 0.010 | | .003 | | -0.052 | | -0.011 |
| PA_T1_ 🡪 NA_T2_ | -0.024 | | 0.011 | | .031 | | -0.046 | | -0.002 | | -0.029 | | 0.011 | | .008 | | -0.050 | | -0.007 |
| Variance Explained (*R^2^*) |  |  | |  | |  | |  | |  | |  | |  | |  | |  | |
| Value_T2_ | 0.003 | |  | |  | |  | |  | | 0.001 | |  | |  | |  | |  |
| PA_T2_ | 0.013 | |  | |  | |  | |  | | 0.014 | |  | |  | |  | |  |
| NA_T2_ | 0.011 | |  | |  | |  | |  | | 0.011 | |  | |  | |  | |  |

*Note*. Each Cross-lagged panel model was fitted for one higher order value and the two affect types. PA: Positive Affect; NA: Negative Affect.
